# A Dickson polynomial based group key agreement authentication scheme for ensuring conditional privacy preservation and traceability in VANETs

**DOI:** 10.1038/s41598-025-89208-5

**Published:** 2025-02-20

**Authors:** Y. Rajkumar, S. V. N. Santhosh Kumar

**Affiliations:** School of Computer Science Engineering and Information Systems (SCORE), Vellore Institute of Technology, Vellore, Tamil Nadu India

**Keywords:** Dickson polynomial, Conditional privacy preservation authentication (CPPA), Chinese remainder theorem (CRT), Group key agreement (GKA), Vehicular adhoc networks (VANETs), Energy science and technology, Engineering, Mathematics and computing

## Abstract

VANETs exchange data in highly dynamic open wireless access environments which are prone to security and privacy attacks. In order to safeguard the transmitted data, group key agreement authentication (GKAA) technique is used. Utilization of group key allows entities to corroborate a group key for secure VANET communication in an unsecure wireless communication channels. The traditional GKAA consumes a considerable amount of resources, verification delay is very high. Since the group key is computed and administered solely by TTA, it leads to central tendency. Additionally the communication delay soars high. To alleviate the problems of computational cost, communication cost, security, conditional privacy, central tendency, a Dickson polynomial based conditional privacy preservation authentication based on group key authentication (GKA) for VANETs has been proposed. The proposed work involves the use of Dickson polynomial to improve the security strength of TTA while authentication vehicles. Since it is based on chaotic mapping algorithm, wherein the chaotic map provides a one-way hash function; Dickson polynomial is used to corroborate a publicly distributed group key; it alleviates the complex modular or scalar multiplication performed using Elliptic curves. The group key gets computed in a distributed fashion by using the Chinese Remainder Theorem (CRT) and gets updated dynamically without the aid of TTA. Conditional Privacy has been ensured by the tracing back the pseudonyms in case of any illicit behavior exhibited by the vehicles. The proposed scheme is lightweight and lowers the communication, computation cost involved during authentication and verification. Performance analysis has been carried out by using BAN logic and ROR model thereby ensuring the security and efficiency. Thus the proposed authentication technique outperforms the traditional certificate-less and group key authentication schemes in terms of improvement in computation cost of 39%, communication cost of 672 bits for a single message with a less verification delay.

## Introduction

Conventional transportation systems (CTSs) suffer from problems such as traffic congestion, traffic delays, traffic accidents, economical loss, infrastructure damage and deaths^1^. To cater these problems of traffic, ITS emerged as a solution to mitigate the traffic problems encountered by the traditional transportation^2^. VANETs act as a core functioning entity of ITS^3^. Vehicles, especially cars have now been equipped with sensors and information computing facilities along with the information and communication technologies (ICT) opened a new arena of applications revolutionizing the modern transportation systems^4^. VANETs are applied in a wide variety of applications relating to advanced cruise control, vehicle collision avoidance, cooperative and autonomous driving^5^. Vehicles are capable of forming a network on-the-go referred to as VANETs. They are highly dynamic and self-organized in nature^6^. The major advantage of the VANETs is to provide the safety, efficiency and comfort to the commuters^7^.

Data transmissions in VANETs are highly subjugated by different kinds of security and privacy concerns^8^. Since the data gets exchanged through a wide open wireless communication channel, it is highly susceptible to message modification, tampering, impersonation, signature forgery, and side channel attacks^9^. Thus it provokes a hasty concern to protect the data that has been exchanged through the vehicles on the roads to enable a secure and a sustainable ITS. Traffic information being trapped or hijacked by an intruder may manipulate, modify or de-route the commuter leading to unforeseen consequences. The leakage of the sensitive data during the transit may also be viable which questions the integrity and the legality of the data being transmitted. Hence anonymity of data has to be ensured^10^. In case of medical emergencies, patient information on roads falling into the hands of intruder may tend to critical situations of life loss, delay in treatment or even medical errors^11^. Similarly, an invader having access to the personal travelling preference may have the possibility to exploit in any possible way. It is essential to preserve the privacy by means of pseudonyms. Usage of pseudonyms helps the signature verifier for tracking the original identification of the vehicles that involve in adverse illegal activities^12^. There is an exclusive need to process the large amount of messages being generated which may lead high computation delay or processing delays. Processing large amounts of messages by the RSUs may be cumbersome, overloading resulting in attrition, exposed, physical side-channel attacks, replication attacks, DoS attacks and TPD attacks in the network^13^. This consequence will lead to the decline in the performance of the network. In order to achieve security and privacy several authentication schemes relating to public key-based^14^, identity-based^15^, pseudonym-based^16^, certificate-less cryptography^17,18^ have been proposed. Most of these cryptographic schemes utilize either bilinear pairing or elliptic curves in order to ensure the security of the data being generated and transferred. Though ECC is 20 times faster than the bilinear pairing operation, it still suffers from lengthy signatures which degrade the computation efficiency and storage overhead^19^.

To alleviate these problems, it is desired to design a lightweight distributed authentication scheme by using Dickson polynomials has been proposed^20^. The proposed scheme utilizes lightweight mechanisms such as XOR operations and chaotic maps^21^ to ensure security and high connectivity among the vehicles in the network. The authentication is lightweight and employs Chinese Remainder Theorem for the dynamic distribution of group keys. Our method involves the following advantages. First, it effectively distinct security objectives and resists message modification, replay, impersonation and so on. Second, it reduces the computational overhead incurred with the cryptographic methods based on ECC or bilinear pairing which means that it performs distributed authentication.

## Research gaps

Existing authentication schemes in VANETs suffers from problems such as central point of failure, traceability and privacy preservation. In addition, when the pseudonyms are stored inside the TPDs of the OBUs, there is a high possibility of TPD attack. TTA faces high computational burden when it performs message signature verification and revocation in a centralized manner. Chebyshev polynomial based conditional privacy preservation authentication scheme though it alleviates the aforementioned problems it suffers Bergamo et al.^22^ attack. These challenges provide a motivation to design a conditional privacy preservation authentication scheme that would be distributed, traceable, revocation and thereby achieving conditional privacy preservation in VANETs.

### Major contributions

The major contributions of the proposed authentication scheme are outlined as follows:

- A new distributed group key agreement authentication scheme has been proposed to ensure conditional privacy preservation implemented using Dickson polynomial in lieu of ECC or Bilinear Pairing with reduced computation and communication overheads.
- The proposed scheme is lightweight, distributed and performs dynamic distribution of group keys using Chinese Remainder Theorem (CRT) where the vehicles are managed efficiently while stepping in or out of a group thereby achieving V2V and V2I communications
- The proposed scheme achieves location, user, conditional privacy, traceability and resists replay, message modification and impersonation attacks, TPD and Bergamo et al.^22^ attacks being encountered.
- The proposed scheme utilizes group key authentication to reduce the computational burden incurred by the TTA while performing verification by making it distributed. This alleviates the central dependency problem associated with the conventional authentication schemes.
- A formal security analysis has been carried out by using both BAN logic and ROR model to demonstrate the security of the proposed scheme. Performance analysis has also been carried out by using performance metrics such as computation, communication, storage costs and verification delay.

### Organization of the article

The manuscript has been structured as follows: “Introduction” Section encompasses the introductory segment, while “Related Works” Section delves into the comprehensive examination of the research endeavors pertaining to group key authentication techniques. “Preliminaries” Section introduces the foundational elements, system design, and security objectives that serve as the foundation for the proposed distributed authentication scheme. “Proposed Methodology” Section elaborates on the methodology of the authentication scheme. “Security Analysis” Section presents both the formal and informal security analysis. “Experimental Setup and Performance Metrics” Section offers a performance evaluation of the proposed authentication scheme, including benchmarks. Finally, “Results Discussion and Analysis” Section concludes the paper. Table 1 presents a compilation of abbreviations employed in the present study.

**Table 1:** Abbreviations utilized in our Survey.

Acronym	Description
VANET	Vehicular Adhoc Network
CTS	Conventional Transportations System
ICT	Information and Communication Technology
ITS	Intelligent Transportation Systems
CRT	Chinese Remainder Theorem
BAN	Burrows-Abadi-Needham
DSRC	Dedicated Short-Range Communication
DoS	Denial-of-Service
IEEE	Institute of Electrical And Electronics Engineers
ECC	Elliptic Curve Cryptography
TTA	Trusted Transport Authority
OBUs	On-Board Units
RSUs	Road-Side Units
AS	Authentication Server
KGC	Key Generation Center
V2V	Vehicle-to-Vehicle
V2I	Vehicle-to-Infrastructure
CMCDHP	Chaotic Maps based Computational Diffie Hellman Problem
GF	Galois Field
MitM	Man-in-the-Middle
DoS	Denial-of-Service
ROR	Random Oracle Model
XOR	Exclusive-OR operation
CMCDHP	Chaotic-Map based Computational Diffie-Hellman Problem
EAP	Extensible Authentication Protocol
WAVE	Wireless access for Vehicular Environment
GPS	Global Positioning System
TPD, IIoTs	Tamper-Proof Device, Industrial Internet of Things
MIRACL C++	Multi-precision Integer and Rational Arithmetic C/C++ Library
RAM, C-V2X	Random Access Memory; Connected-Vehicle-to-Everything
LTS, IoVs	Long-Term Support, Internet of Vehicles
SUMO	Simulation of Urban Mobility
OMNET++	Objective Modular Network Testbed in C++
PUF	Physically Unclonable Functions
ESL	Ephemeral Secret Key Leakage
AVISPA	Automated Validation of Internet Security Protocols and Applications
CPPA	Conditional Privacy Preservation Authentication
MitM	Man-in-the-Middle
GKA	Group Key Authentication
PKC	Public Key Cryptography
CRT	Chinese Remainder Theorem
GKAA	Group Key Agreement Authentication
GCD	Greatest Common Divisor
MAC	Message Authentication Code
EAP	Extensible Authentication Protocol
ECC	Elliptic Curve Cryptography
ESL	Ephemeral Secret-Key Leakage
CLAS	Certificate-less Aggregate Signature
CPPA	Conditional Privacy Preservation and Authentication
CMDDHP	Chaotic Map Dickson Diffie-Hellman problem
VCS	Vehicular Communication System
5G; EUF-CMA	Fifth Generation; Existential Unforgeability under chosen message attacks
HSMs	Hardware security module
SSK	Secure Session Key
MAC	Medium Access Control

## Related works

Various Authors have proposed numerous mechanisms to counter the issues related to security and privacy by designing numerous authentication schemes. To achieve security, conditional privacy and to improve the efficiency schemes such as certificate-based, identity-based, pseudonym-based, certificate-less are proposed. This section provides a brief understanding of some of the related works pertaining to authentication schemes.

### Conditional privacy preserving authentication schemes (CPPAs)

CPPA ensures security, anonymity and traceability in the network. Upon any malfunctioning, CPPA provides efficient tracing back of vehicles with the help of pseudonyms stored inside the TPDs of the vehicles. Several schemes based on conditional privacy preservation have been proposed by various academicians around the globe. Among them, Liang et al.^23^ proposed an authentication scheme to address the problem of ESL from the storage devices by utilizing PUFs. Their system model also addressed the problem of computational overhead soars due to frequent contact with the vehicles in motion. Their proposed authentication scheme utilized PUFs to achieve conditional privacy authenticated key agreement for VANETs. The proposed PUF based mutual authentication between the vehicles and RSUs does not require the participation of TA. Utilization of pseudonym and conditional privacy provides anonymity and traceability in case of malevolent vehicles. The proposed protocol is resilient against cloning, physical, vehicle-RSU impersonation, MiTM, replay, ESL and known session key attacks. However, the computation and communication cost soars high which needs to be reduced. Yang et al.^24^ has come up with a secure, pairing-free, energy-efficient CLAS scheme which ensures CPPA for VANETs. Their proposed scheme addresses the problem of coalition attacks from malicious RSUs and public-key replacement attacks from malicious vehicles. Their proposed scheme involves ECC based authentication scheme which provides anonymity of vehicle identity and traceability of malevolent vehicles. Their methodology is secure against public key replacement, forgery, and tampering, malicious KGC, coalition attacks and lowers the computation and communication costs efficiently. Roy et al.^25^ has come up with a novel multiple TA model for fog-enables VANETs to address the problems of centralized trusted authority, single-point-of-failure, service unavailability due to increased traffic load. Their proposed model involves multiple TAs where the traffic processing load has been distributed across many sub-TAs. Their model also incorporates a fog node which takes care of the managing the RSUs centrally eliminating the need to perform individual authentication of RSUs. Their proposed protocol involves CPPA ensuring security and privacy it suffers from high computation and communication costs. Additionally utilized TA may behave as malevolent which weakens the security strength of the proposed scheme. Zhu et al.^26^ proposed a CLAS based authentication scheme based on ECC. Their proposed system utilized ECC based multiplication, one-way hash functions, XoR operation and dynamic pseudonyms in order to guarantee security and privacy. However the proposed protocol suffers from high computational overhead. TA incurs additional overhead in maintaining subsequent updation of pseudonyms. Zhang et al.^27^ proposed a novel authentication scheme based on HSMs to address the problem of certificate storage. In order to lower the computation costs, forward secrecy, a lightweight authentication scheme that ensures CPPA has been proposed. Their scheme involves SSK updation which involves identity-based batch multi-signature algorithm which ensures traceability and revocation. Though the proposed work reduces the computational complexity to a considerable extent, it still suffers from communication overhead due to frequent secret session key updates. Wang et al.^28^ has come up with a CPPA ensure anonymity and conditional privacy. The proposed authentication scheme has been designed without pseudonyms and is resilient to linkage attacks. Their proposed scheme is efficient and achieves anonymity, unlinkability, non-repudiation, message integrity, traceability and revocation. Though their proposed scheme is computationally efficient it is suitable only for V2V communications. Roy et al.^29^ has come up with an authentication protocol which has backed up by the elimination of TA ensuring zero-trust mechanism for VCS. Their scheme addressed the problem of communication delay, central tendency and DoS with the help of 5G connectivity. Their system has utilized ECC and Secret sharing scheme to ensure the security against vehicles and RSUs and provides support for both V2V and V2I communications. However the authentication delay is still high which needs to get reduced. Kumar et al.^30^ proposed a (2,n) threshold secret sharing scheme in combination with ECC to provide solution to the problem of computational overhead, authentication delay and re-authentication. Their proposed scheme eliminated the use of TA and subsequently reduces the communication overhead. The major novelty is that atleast two of n registered vehicles cooperation is required to compute a shared secret key which is highly not possible for an attacker to counter. Though the proposed scheme ensures security and privacy, the computation and the communication cost is higher in case of V2I communications. Moni et al.^31^ proposed a pseudonym-based privacy preservation scheme to address the problem of traceability incurred in processing the certificate revocation lists. Though it reduces the overhead it suffers from storage cost in maintaining the cuckoo filter and is not suited for V2I communications. Samra et al.^32^ has come up with a CPPA scheme based on ECC and ring signatures. Though the scheme achieves traceability it suffers from computation overhead and non-repudiation. Wang et al.^33^ proposed a certificate-less conditional privacy preservation scheme based on Bilinear pairing. Their work has achieved full aggregation and reduced the communication cost, it suffers from high computation cost due to pairing operation. Wang et al.^34^ has come up with a certificate-less CPPA to ensure unlinkability, anonymity and to lower the computational and computational overhead incurred in VANETs. Their proposed scheme involves the use of Bilinear pairing technique and ensured traceability, anonymity. Though it is resilient against reply, impersonation, message modifications and MiTM attacks it has to be tested and validated for real-time implementations. Xiong et al.^35^ proposed a mutual authentication protocol to ensure conditional privacy, anonymity and traceability in VANETs to address the problem of forward and backward secrecy. Their proposed protocol utilized bilinear maps and puncturable authentication along with parallel key insulation technique which eliminates the computational overhead incurred when authentication involves RSUs. However the authentication delay is very high.

### Group key agreement authentication schemes (GKAs)

Group key Agreement authentication schemes have been proposed to reduce the computational overhead incurred by the TTA thereby eliminating the central point of failure. To ensure security and conditional privacy several GKAA schemes have been proposed. Among them, Ali et al.^36^ has proposed group shared key authentication scheme based on ECC and a combiner for hash function to provide security in case of IIoTs. Their proposed scheme utilized group secret keys between the industrial objects. Digital signatures are utilized and the scheme is highly resilient against attacks such as brute force, stolen verifier table, device capture, MiTM, Cipher-text, key attacks. However their protocol needs to be verified for computational and communication overheads. Zhan et al.^37^ proposed a secure group authentication scheme where the GKA are updated based on condition matching to ensure conditional privacy in VANETs. Their proposed authentication scheme involves the use of ECC and fog-computing for VANETs in order to reduce the problem of computational overhead and key management burdens. The scheme achieves cross-domain authentication, anonymity, traceability, key-escrow freeness, perfect forward secrecy and is resistant to attacks such as replay, impersonation and modification attacks. However, their schemes incur high computation and communication costs which need to get reduced. Li et al.^38^ has come up with a MAC based group key authentication scheme to achieve anonymity and conditional privacy. The scheme lags in key update mechanism and is vulnerable to security attacks. It cannot achieve forward and backward security. Islam et al.^39^ proposed a secure password based GKAA scheme to achieve conditional privacy. Though their work has reduced the computational cost to a greater extent it still suffers from communication overhead since it involves repeated communication with the transport authority. Cui et al.^40^ addressed the problem of security and privacy by proposing a novel dynamic GKA scheme to prevent forgery and session specific key attacks. Though the protocol extremely supports C-V2X application is suffers from high communication overhead which needs to be reduced. paliwal et al.^41^ has come up with a novel multi-party GKA scheme to ensure conditional privacy, and to overcome the data breach during data transmission. Their proposed protocol incurs authentication and communication delay which involve high transmission cost. Xu et al.^42^ addressed the problem of forward secrecy and malicious attacks by proposing a continuous GKA for IoVs. Their main aim is to design a GKA which provide efficiency against collusion attacks. The proposed protocol utilizes treeKEM architecture for group key authentication and management along with threshold secret sharing scheme for encryption. Though their protocol reduces the computation cost it still suffers from communication overhead during the communication and key updation phase. Cui et al.^43^ proposed a conditional privacy preserving GKA using chaotic maps for VANETs. Though their proposed protocol efficiently lowers the computation overhead it is high susceptible to Bergamo et al.^22^ attacks and suffers from high communication cost. Lai et al.^44^ has come up with a three party key agreement authentication scheme utilizing Chebyshev chaotic maps for VANETs. Their scheme suffers from the disadvantage such as bilinear pairing which incurs high computation overhead and is suitable only for V2I communications. Similarly, Lee et al.^45^ utilized Chebyshev chaotic maps based on diffie-hellman assumption. The major advantage is that it reduces the computational complexity incurred in signing and verification. Yang et al.^46^ proposed a Chebyshev polynomial based GKAA scheme to achieve conditional privacy and traceability. However, the proposed scheme is suitable only for V2V communications. The major drawback of the scheme is that it cannot resist Bergamo et al.^22^ attack where the security assumption is based on real numbers and is sensitive to initial value^47,48^. Table 2 provides the comparative analysis of various authentication schemes. From the literary related works, it is apparent that most of the CPPAs either use bilinear pairing or ECC to counter the TPD attacks and non-repudiation. However, these schemes suffer from computational complexity and increased length of signatures. Similarly, the GKAs suffer from high communication cost, forward and backward security. Hence in order to counter these issues, our scheme aims to counter by utilizing pseudonym to achieve non-repudiation, forward and backward security and CRT for key management. Additionally, it is necessary to reduce the computational complexity of the TTA; the scheme is distributed where the computational burden gets reduced. In addition, the key operations and values are also reduced subsequently. This results in the design of a new authentication scheme to achieve conditional privacy preservation and traceability using the Dickson polynomial in VANETs.

**Table 2:** Comparative analysis of various existing authentication schemes.

Ref	Type	Problem addressed	Encryption method	Assumption	Resistance to attacks	Benefits	Limitations	Application
^23^	Pseudonym based CPPA	ESL	PUF, ECC	DLP, CDHP	Cloning, physical, impersonation, MiTM, relay, known session key and ESL attacks	Ensures Mutual authentication, key agreement, conditional privacy	1.Computational cost soars high 2.Temporal Secret leakage persists	VANETs
^24^	Identity based CPPA	Coalition, Public Key replacement, forgery attacks	ECC	DLP	Forgery, tampering, collusion and malicious KGC attacks	Ensures message integrity, authentication, anonymity, traceability, Conditional identity privacy, unlinkability	Computational Overhead is still high	VANETs
^25^	Identity based CPPA	Centralized trusted authority, Single-point-of-failure and Service unavailability	ECC	DLP	Replay attack, Privileged Insider or Stolen verifier table attack, ESL attack and Key escrowness	Ensures anonymity, untraceability, conditional privacy, mutual authentication, Forward/Backward secrecy	1.Computation cost incurred by the parent TA is high 2.Session key attacks weakens the security 3.The stored data can be easily hacked by the TA and perform leakage	Fog-enabled VANETs
^26^	Identity based CPPA	Security, Privacy, Key Escrow Problem	ECC	DLP	Modification, Forgery, Replay and MiTM attacks	Ensures message authentication, Dynamic pseudonym, unlinkability, conditional traceability, forward and backward secrecy	1.Computational cost is high 2.Pseudonym updation is an additional overhead incurred	VANETs
^27^	Identity based CPPA	Forward Secrecy,	HSM	CDHP, BDHE	Replay, Session Key attacks	Ensures identity privacy, traceability, authentication, untraceability	Incurs high communication overhead	VANETs
^28^	Identity based CPPA	Linkage attack due to pseudonyms	Bilinear Pairing	k-CAA	-	Ensures anonymity, unlinkability, non-repudiation, message integrity, traceability and revocation	1. Suited only for V2V communications 2. Suffers from central tendency	VANETs
^29^	Identity based CPPA	Authentication Delay, DoS, Central tendency, Compatability	ECC, Secret Sharing Scheme	Generic	Tampering, Spoofing and DoS attacks	Ensures conditional privacy, repudiation, tampering, information disclosure, Elevation of privilege	Authentication Delay is still very high	VANETs
^30^	Identity based CPPA	Authentication Delay, Computational Overhead	ECC, (2,n) Threshold Scheme	Collision Resistant, In distinguishability in Cipher-Text	ESL, replay, impersonation, RSU compromise, Malev olent Vehicle User, privileged insider/Stolen Smart Card Attacks	Ensures typo detection, privacy, mutual authentication, forward/backward secrecy	Communication cost is very high	NextGenV2V
^31^	Pseudonym based CPPA	Certificate Management Overhead	ECC, Cuckoo Filter, (MHT)	Merkle Hash Tree	MiTM, Impersonation and replay attacks	Ensure mutual authentication, identity anonymity,	Does not support V2I communications	VANETs
^32^	Identity based CPPA	Computation & Communication Overhead	Elliptic Curves and Ring Signatures, Aggregation	CDHP, DDHP, sqCDHP, divCDHP	Impersonation, Signature forgery attacks	Ensures traceability, conditional privacy, message authentication, unforgeability, anonymity and efficiency	1.Computational overhead incurred by the TTA is very high 2.Non-Repudiation cannot be achieved	VANETs
^33^	Pseudonym based CPPA	Large verification delays & high communication Overhead	Bilinear Pairing, Aggregation	CDHP	Replay attack	Ensures message authentication, integrity, Non-Repudiation, anonymity, unlinkability, Traceability	Computational cost is high due to pairing operation	VANETs
^34^	Pseudonym based CPPA	Security, unlinkability & Anonymity	Bilinear Pairing, Batch Authentication	DLP, CDHP	Replay, impersonation, message modification attacks	Ensures message authentication, unlinkability, traceability, anonymity, Revocation	Tested & validation for real-time implementations	VANETs
^35^	Identity based CPPA	Forward & Backward Secrecy	Bilinear Maps, Puncturable Authentication, Parallel Key Insulation	CDHP, eCDHP	Chosen-Message Attacks	Ensures anonymity, mutual authentication, conditional privacy, unlinkability, forward secrecy, backward secrecy	Authentication Delay is very high	IoVs
^36^	Identity based GKA	Security	ECC	CDHP, DLP	Chosen Cipher text, known plaintext, chosen plaintext, key, MiTM, Brute force, device capture, stolen verifier,	Ensures group authentication, message integrity	Has to be validated using real-time implementations	IIoTs
^37^	Pseudonym based CPPA	Cross-domain dynamic group session key negotiation and high Computational overhead	ECC	DLP, CDHP	Replay, impersonation and tampering attacks	Ensures mutual authentication, fog node anonymity, vehicle anonymity, fog node traceability, vehicle traceability, Session key establishment, Cross-domain authenticated key agreement, Traffic condition matching, forward secrecy	Updation of session key invokes high communication overhead	Fog-cloud Based VANETs
^38^	MAC based GKA	Security, privacy	MAC, ECC, Shamir’s (k,n) threshold secret sharing	-	Replay, forgery and message modification attacks	Ensures message authentication, privacy preservation, conditional privacy, unlinkability	Suited only for V2V communications	VANETs
^39^	Identity based GKA	Computational Cost	Password, Hash function, Group Key	–	Replay, Impersonation, message modification, offline-password guessing attacks	Ensures conditional privacy, traceability, message authentication, group-key agreement, backward secrecy, forward secrecy	Authentication delay persists	VANETs
^40^	Identity based GKA	Signature, Certificate forgery,	Bilinear Maps	ECDLP, DBDHP	Replay, forgery MiTM	Ensures group key agreement, mutual authentication, anonymity, traceability, forward secrecy, backward secrecy	High communication Overhead	C-V2X
^41^	Identity based GKS	Data Breach	Multiparty Group Key, Hash algorithm, Threshold Modulo	–	KSSTI attack, impersonation, known key, replay, de-synchronization, message modification, DoS attacks	Achieves unlinkability, mutual authentication, traceability and dynamic session specific modulus, conditional privacy	Communication overhead still persists	VANETs
^42^	Pseudonym based GKA	Data Breach	Dynamic Key Rotation, treeKEM, Threshold secret sharing scheme, Pseudo-random function, Pseudo-random permutation	–	Collusion,	Achieves Conditional privacy, confidentiality, unlinkability, PCFS,	Communication cost has to get reduced	IoVs
^43^	Identity based GKA	Security, Privacy	Chaotic Map, Chebyshev polynomial	DLP, DHP	Replay, Insider attacks	Achieves group authentication, deniability, privacy preservation and forward secrecy	1.Susceptible to Bergamo et al.^22^ attacks 2. Communication cost is high	VANETs
^47^	Identity based GKA	Security, privacy, Computation & Communication Overhead	Chaotic Map, Chebyshev polynomial	eCDLP	Known key, replay, impersonation, MiTM	Achieves mutual authentication, session key security, key agreement, data integrity, perfect forward secrecy,	1.Cannot resist insider attacks 2.Suffers from Bergamo et al. ^22^ attack	Networks
^48^	Identity based GKA	Security, privacy, Computation & Communication Overhead	Chaotic Map, Chebyshev polynomial	CDHP	Replay, Modification	Achieves group authentication, conditional privacy, location privacy, traceability, forward and backward secrecy	Suffers from Bergamo et al. ^22^ attack	VANETs

## Preliminaries

This chapter provides an overview of the context, concept and system design employed in the development of the proposed distributed authentication technique for vehicular ad hoc networks.

### Dickson polynomial

Due to the irreducible property, polynomials find its applications in security and authentication^49^. Polynomial cryptography exhibits two different properties namely semi-group and irreducibility^50^. Utilization of Dickson polynomial offers security against TPD and ephemeral key attacks it finds application in key agreement schemes. Due to the irresistive nature of Chebyshev polynomial towards MiTM and Bergamo et al. attack Dickson polynomial has been adopted^51–55^. Dickson polynomials are defined over the finite field recursively which depends upon the parameter $n$ and $a$ mapping the elements bijectively which makes it highly suitable for low resource-constrained environment like VANETs^56^

*Dickson Polynomial and its properties:* Dickson Polynomial^20^ for a variable ${\text{a}} \in {\text{F}}_{{\text{q}}}$ defined by an integer n; then the Dickson Polynomial ${\text{D}}_{{\text{n}}} \left( {{\text{x}},{\text{ a}}} \right)$ over any finite field ${\text{F}}_{{\text{q}}}$ can be defined as

1 $$D_{n} \left( {x,a} \right) = \mathop \sum \limits_{i = 0}^{\frac{n}{2}} \frac{n}{n - i}\left( {\begin{array}{*{20}c} {n - i} \\ i \\ \end{array} } \right)\left( { - a} \right)^{i} x^{n - 2i}$$

where $\frac{n}{2}$ is the largest integer $\ge \frac{n}{2}$. The Dickson Polynomials gets gratified by the continuous relation such that $D_{n} \left( {x,\alpha } \right) = x D_{n - 1} \left( {x,\alpha } \right) - \alpha D_{n - 2} \left( {x,\alpha } \right); n{ \succcurlyeq }2.$ Under the initial condition ${\text{D}}_{0} ({\text{x}},{\upalpha }$) = 2 and ${\text{D}}_{1} ({\text{x}},{\upalpha }$) = x and some of the other polynomials are computed by using the Eqs. (2–5) as follows:

2 $$D_{2} \left( {x,\alpha } \right) = x^{2} - 2\alpha$$

3 $$D_{3} \left( {x,\alpha } \right) = x^{2} - 3\alpha$$

4 $$D_{4} \left( {x,\alpha } \right) = x^{4} - 4\alpha x^{2} + 2 \alpha^{2}$$

5 $$D_{5} \left( {x,\alpha } \right) = x^{5} - 45\alpha x^{3} + 5 \alpha^{2} x$$

The most important characteristic property of the Dickson polynomial is that is satisfies commutativity under composition *when* $\alpha$ = *0 or 1.*

Definition 1: Semi-group Property: Dickson polynomials exhibit semi-group property under composition which can be defined as follows:where p is a prime number. Hence

$$D_{n} \left( x \right) \equiv \left( {2xD_{n - 1} \left( x \right) - D_{n - 2} \left( x \right)} \right) mod p; \forall n \ge 2;x \in \left( { - \infty , + \infty } \right);$$

6 $$\begin{aligned} D_{mn} \left( {x,1} \right) & = D_{m} \left( {D_{n} \left( {x,1} \right),1} \right) \\ & = D_{m} \left( {x,1} \right) o D_{n} \left( {x,1} \right) \\ & = D_{n} \left( {x,1} \right)o\;D_{m} \left( {x,1} \right) \\ & = D_{n} \left( {D_{m} \left( {x,1} \right), 1} \right) \\ & = D_{nm} \left( {x,1} \right) \\ \end{aligned}$$

The semi-group property exhibited by the Dickson polynomials is an essential property and finds a wide variety of cryptographic applications which is given by the Eq. (6).

Definition 2: Chaotic-Map based Dickson Diffie-Hellman Problem (CMDDHP): For any given value of x; where $x \in \left( { - \infty ,\user2{ } + \infty } \right)$; $D_{u} \left( x \right)mod p, D_{v} \left( x \right)mod p$ for a large prime p. It is intransigent to compute $D_{uv} \left( x \right)mod p$^47^*.*

### Chaotic map based hash function

Due to the properties of variable-dependency and abstract correlation; chaotic maps are utilized to build the hash functions. One notable benefit of utilizing chaotic hash is its ability to streamline normal procedures. Chaotic Hash function^57,58^ used along with Dickson-based sequences alleviates the problem of computational overhead and storage complexity. This results in increase in the performance of the OBUs. The algorithm works according to^46^ which consist of both the input and the output as follows:

**Input:** A discrete length of a string of bits which is of length y.

**Output:** Chaotic Hash Value of 128 bits.

### Chinese remainder theorem (CRT)

CRT asserts that, under the condition that the divisors are pairwise co-prime, it is feasible to determine the remainder of an integer divided by its product in a unique manner, provided that the remainders resulting from the Euclidean division of n by multiple integers are known^23^.

CRT can be stated as follows:

Let $\left\{ {l_{1} , l_{2} ,l_{3} , \ldots , l_{n} } \right\}$ be the set of positive integers which are relatively prime to one another;

Let $\left\{ {b_{1} ,b_{2} ,b_{3} , \ldots ,b_{n} } \right\}$ be the set of positive integers. Therefore the pair of congruences given by the chinese remainder theorem can be defined as.

{$Z \equiv b_{1} mod l_{1}$; $Z \equiv b_{2} mod l_{2} , \ldots ,Z \equiv b_{n} mod l_{n}$} gives an individual solution defined by $mod \partial_{h} = \mathop \prod \limits_{i = 1}^{n} \left( {l_{i} } \right)$. The trusted transport authority finds the solution based on the Eq. (7)

7 $$Z = \left( {\mathop \sum \limits_{i = 1}^{n} \delta_{i} \theta_{i} \vartheta_{i} } \right) mod \partial_{h}$$

where $$\theta_{i} = \frac{{\partial_{h} }}{{l_{i} }}$$ and $$\theta_{i} \vartheta_{i} \equiv 1 mod l_{i}$$.

### System design

**Fig. 1:**
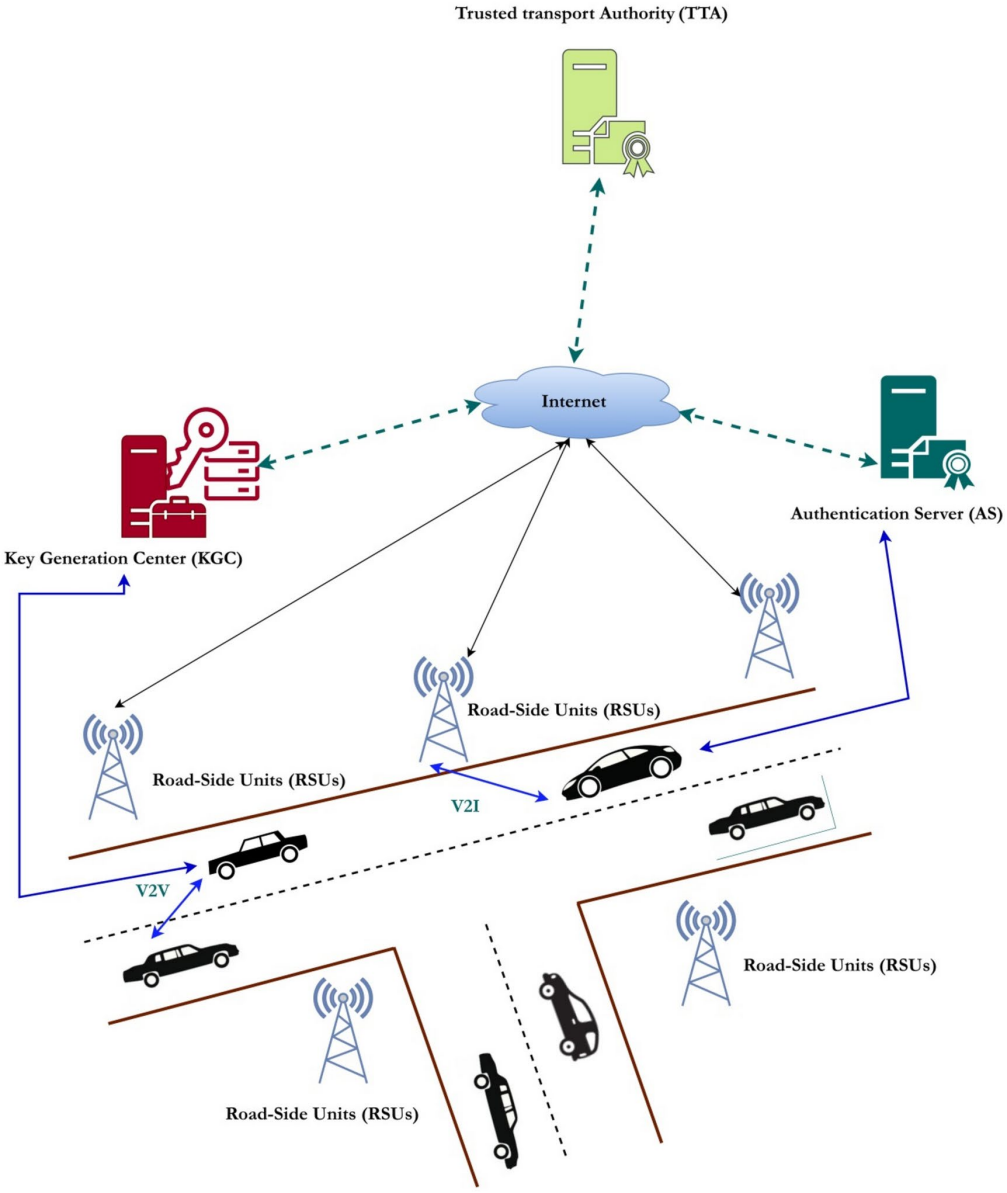
Distributed Architecture of a Typical VANET.

**Fig. 2:**
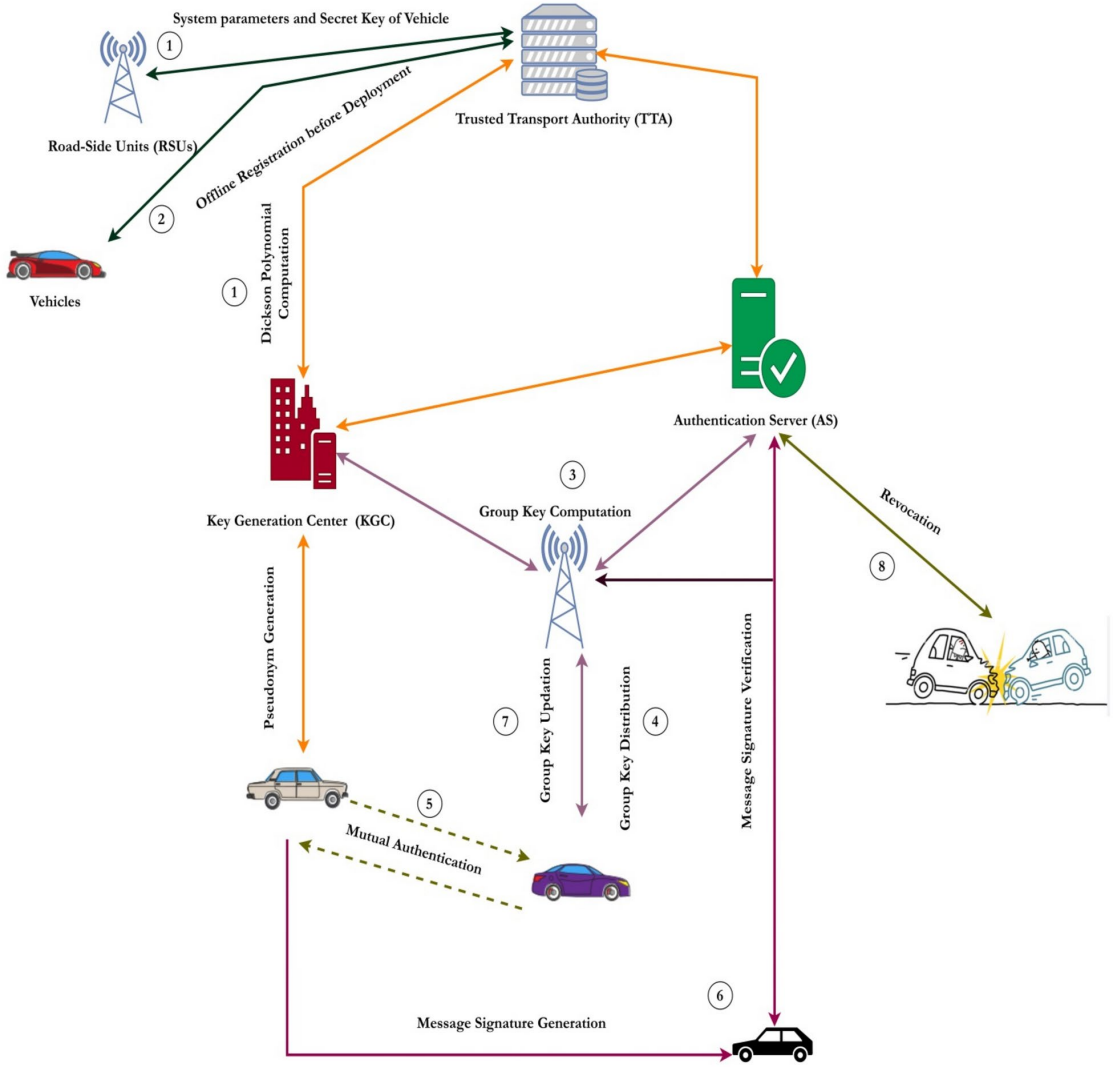
Distributed Working Model.

The typical architecture of a conventional VANET comprises of essential components namely the trusted transport authority (TTA), which includes the Key Generation Center (KGC) and the Authentication Server (AS), as well as the Road-Side Units (RSUs) and the On-Board Units (OBUs). The logical description of these components is achieved through the utilization of three levels, specifically referred to as the upper layer, the middle layer, and the bottom layer. The uppermost tier of the architecture comprises of the TTA, the KGC, and the AS while the intermediate layers comprises of RSUs. The lowest layer is comprised of the vehicles. The exchange of information among these three constituents is enabled by an IEEE standard, namely IEEE 802.11p, which is commonly referred to as DSRC. RSUs serve as an intermediary entity that facilitates communication and coordination between the TTA, KGC, and OBUs. The architectural structure adheres to a hierarchical design in which data transmission is authenticated through the utilization of two private keys generated by both the key generation center and the trusted transport authority. The concatenation of these partial private keys serves as a shield against forging, replay, impersonation, and various other forms of attacks. To compromise a specific message, an intruder must possess two partial private keys and one secret key of the vehicle, hence rendering it highly resistant to key ephemeral assaults. The distributed nature of the VANET model employed in our proposed system is illustrated in Fig. 1. Figure 2 depicts the operational framework of the proposed system.

#### Trusted transport authority (TTA)

TTA is mainly responsible for the initialization, functioning and generation of the public parameters required for the system. TTA is highly trustworthy and it cannot be compromised. The communication between the TTA and the AS can be accommodated wirelessly by means of EAP^59–61^. This protocol takes the responsibility of the vehicle registration, RSUs and AS. Furthermore, legal measures are implemented to address the issue of vehicle impersonation or masquerading, with the capability to trace the true identity of the vehicle in question during any legal procedures. This guarantees the retention of conditional privacy within the system.

#### Key generation center (KGC)

KGC is a separate entity which exhibits functionality such as generation of partial private key for secure message communications. KGC possess enough storage and processing capabilities which administers the role of managing and storing the keys. KGC is fully trusted and cannot be compromised^37^.

#### Authentication server (AS)

AS performs the verification of messages received from the vehicle or the RSUs. Both KGC and AS shares the same database that contains keys. AS stores all the traffic information in the form of database records and performs analytics very often. It is fully trusted and cannot be compromised^39^.

#### Road-side units (RSUs)

RSUs are mainly responsible for the communication between the OBUs and the Upper layered components. RSUs are installed for every 500 m and they are responsible for the message signature verification. RSUs are partially-trusted and are authenticated during the installation of the system by the TTA. RSUs are equipped with sensor device, servicing platform and a V2I communication platform. Utilization of RSUs makes it possible to achieve both V2V and V2I communications.

#### On-board units (OBUs)

OBUs are the vehicles which are registered with the TTA in an offline manner. After registration, each vehicle gets installed with the public parameters by taking the private aspects of each vehicle. Each vehicle is fitted with a TPD called as OBUs. Vehicles communicate with each other with the help of these on-board units. Each vehicle is fitted with communication systems called GPS and sensors for navigation which is responsible for the transmission and record-keeping of highly crucial information like messages and private keys.

### Adversary model

The adversarial model for the proposed authentication scheme is simulated in the form of a game between an adversary $\zeta_{1}$ and the rival $\eta_{1}$. The adversarial model of existential unforgeability against chosen-message attacks (EUF-CMA) can be defined as follows:

*Initialization*: The system initialization algorithm is executed by the rival $\eta_{1}$ in order to generate the system public parameters $pms.$ The public parameters are then sent to the adversary $\zeta_{1}$ and the rival $\eta_{1}$ in a secret way thereby seizing the master secret key $o$.

*Queries*: The adversary $\zeta_{1}$ attempts to query on messages selected by the adversary in an adaptive way. In order to execute a signature query on the message $M_{i}$, then the rival performs the execution of the message signature generation algorithm in order to compute the message signature $\sigma_{i}$ and dispatches it to the adversary $\zeta_{1}$.

*Forgery*: The adversary $\zeta_{1}$ returns a signature which is forged $\sigma_{i}^{\prime }$ on a particular message $M_{i}^{\prime }$ and succeeds in the game iff.

- $\sigma_{i}^{\prime }$ is the legitimate signature pertaining to the message $M_{i}^{\prime }$
- During the querying phase, queries are not performed for the message signature $\sigma_{i}^{\prime }$

The major benefit ${\Im }$ in succeeding the game indicates the generation of a valid forged signature. When ${\Im }$ is trivial for any polynomial adversary $\zeta_{1}$ then the proposed authentication scheme is secure.

### Security goals

Data transmission in VANETs has to be secured. In the proposed work, the group key agreement authentication scheme should satisfy the security properties such as message authentication, data integrity, perfect forward secrecy, perfect backward secrecy, conditional privacy preservation, traceability, unlinkability, anonymity.

#### Data integrity and message authentication

The received data should not be tampered, modified but preventing the data loss and leaks, errors and unauthorized access. The vehicle must be authenticated before it acquires the group key for message transmission.

#### Perfect forward secrecy

Upon any attack, even if the current session key gets compromised, the attacker should not gain any access to the previous session key utilized for Vehicular communications.

#### Perfect backward secrecy

Ensuring that the attackers cannot gain access to future session keys even if the current session key gets compromised during vehicular communications.

#### Conditional privacy preservation

The attacker should not be able to access or break into the original identity of the vehicle involved in communication. In case of legal proceeding TTA or AS performs revocation.

#### Traceability

Upon any illegal activity, it is necessary to trace and revoke back the original identity of the vehicle by the TTA or AS.

#### Unlinkability

Only TTA possess the capability to performs the linking of messages sent from the same vehicle.

#### Anonymity

The original identity of the entities such as $V_{{ID_{i} }}$ and $RSU_{{ID_{i} }}$ cannot disclose their original identity thereby ensuring the anonymity with the help of the pseudonyms.

#### Non-repudiation

The sender cannot deny the sending of the message.

#### Mutual authentication

**A**ll the entities involved inside the communication should verify the legitimacy of each other during the mutual authentication and group key agreement (GKA) phase.

#### Resistance to attacks

The proposed scheme must be resistant to attacks such as replay, message modification, impersonation, coalition, Man-in-the-Middle, Key Exposure, DoS and ESL attacks.

## Proposed methodology

The proposed distributed authentication scheme’s primary objective is to provide conditional privacy preservation in VANET communications by facilitating efficient group key agreement authentication. It is composed mainly of eight phases namely 1. System Initialization Phase 2. Offline Registration Phase 3. Group Key Computation Phase 4. Group Key Distribution Phase 5. Mutual Authentication Phase 6. Message Signing and Verification Phase 7. Group Key Updation Phase and 8. Pseudonym Updation Phase. The first module to be created is called "System Initialization," and its primary job is to generate crucial parameters necessary for the system’s initial operation. Additionally the group key is computed during this phase. In the second phase, known as offline registration, vehicles and RSUs are added to the system and given public and private keys for use in further communications. The group key distribution section is the third component. Here, the TTA must produce the group key that will be used later in the process to authenticate and verify messages.

The fourth phase, known as Group Key distribution phase is responsible for concealing the true identities of vehicles and roadside units behind fictitious names, or pseudonyms. The mutual authentication process is the fifth and the sixth module. The vehicles now have the group key and can reliably communicate with each other while still within range of the roadside equipment. The sixth stage is the group key updation phase. In this step, the group key is updated anytime a vehicle joins or departs the group. The seventh section deals with the generation and signature of messages. In this step, vehicles collect data on the road’s state and construct a time stamped message that must be sent to other nearby vehicles or roadside equipment. The eighth modules consist of the pseudonym updation phase. Table 3 provides the description of the symbols utilized in our proposed scheme.

**Table 3:** List of Symbols.

Symbols	Description
Z	Field Range
o, n, p, q	O, n—Random positive Integer; p-Modulo exponentiation; q-Odd Prime Number
y,$D_{n} \left( y \right)$	y-Random Integer;$D_{n} \left( y \right) - Dickson polynomial of a random integer y$
$GF\left( p \right),$	Galois Field of Prime Integers
$DSK_{i}$	Secret Group Key
$\aleph_{group}$	Final Aggregated Group Key
$\delta_{i}$	Storing place for two variables
${\complement }$	Storage variable for Group Keys
H	Chaotic Map Hash Function
$ASID_{i}$	Authentication Server Identity
$V_{{ID_{i} }}$	Vehicular Identity of a particular vehicle I
$RSU_{{ID_{i} }} ,RSU_{loc}$	Identity of a particular road-side unit I; location of a particular road-side unit
TTA, KGC, OBU	Trusted transport Authority; Key Generation Center; On-Board Unit
$D_{{n_{i} }} \left( {m_{i} } \right)$	Dickson polynomial private key of a RSU
$\left\{ {m_{i } , D_{{n_{i} }} \left( {m_{i} } \right)} \right\}$	Dickson polynomial public key of a RSU
$S_{{RSU_{i} }}$	Secret key of the road-side unit i
$g_{i}$	Newly computed dickson group key
$\Delta T_{i}$	Timestamp i
$kpvk_{i}$	Key Generation center private key
$D_{{\vartheta_{i,n} }} \left( y \right)$	Dickson polynomial of a vehicle computed by using a random positive integer y
$PSID_{{V_{i,n} }}$	Pseudonym identity for each vehicle
$TTS_{{V_{i} }}$	Timestamp value of a vehicle i
$M_{{V_{i} }}$	Message generated or broadcasted for a vehicle V_i_
$Enc_{i}$	Encrypted text
$\sigma_{i}$	Message Signature

### System initialization phase

TTA initiates the system initialization phase. TTA computes the field of prime numbers over the galois field by selecting an odd prime number and a random positive integer. Additionally, TTA chooses a system secret key for the whole system from the multiplicative field. To compute a Dickson polynomial, KGC selects a random number from the Galois field of a selected prime number. Following the selection of a random number, KGC selects a secret key for each car and RSU that will be given out at the time of vehicle registration and forwards it to the TTA. Algorithm 1 provides the steps involved in the system initialization phase.

**Algorithm 1:**
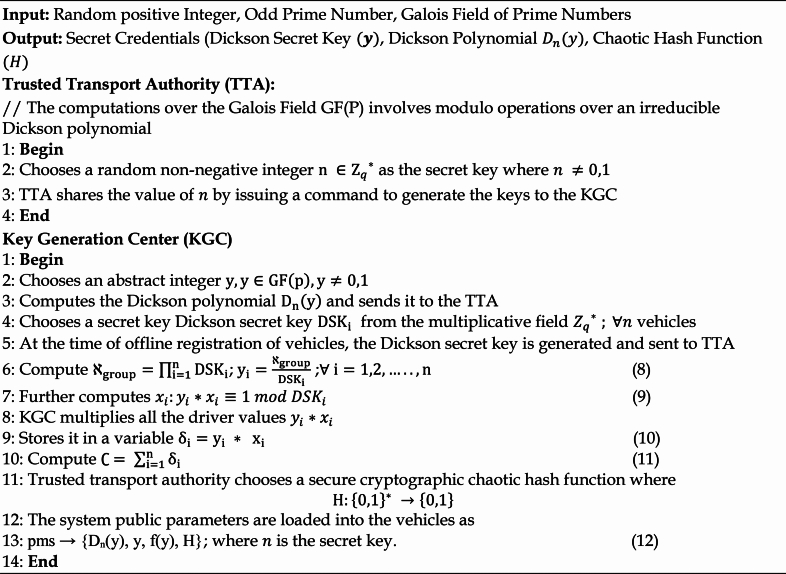
System Initialization Phase

### Offline registration phase

#### Offline vehicle registration phase

Each vehicle and RSU is securely registered offline with the TTA before to the particular system’s operation. The personal credentials such as name, address, phone number, email address, car number, type, and Iris biometric data are all submitted by each owner of a vehicle. Following TTA registration, KGC generates a special secret Dickson group key for every car and sends it to that vehicle. The procedures to be taken during the offline registration phase are listed in Algorithm 2. A duplicate of the same is then kept in the AS’s tracking database. The third step illustrates how the identification and secret key of the matching vehicle are kept in the tracking database.

**Algorithm 2:**
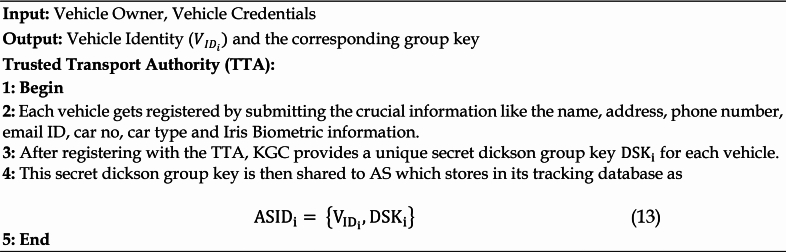
Offline Vehicle Registration Phase

#### Offline RSU registration phase

The RSUs are registered with the TTA prior to the RSUs being installed. The first step illustrates how the location and identity of the RSUs are obtained during registration. Following verification, KGC computes the Dickson polynomial and the Dickson secret key, which serve as the corresponding RSU’s public key and are represented in step 4. Algorithm 3 provides the steps necessary for offline RSU registration phase. Next, KGC selects the RSU secret key that has been transmitted to it. A duplicate of the secret key will be kept in the KGC database, as indicated by equation in the final step.

**Algorithm 3:**
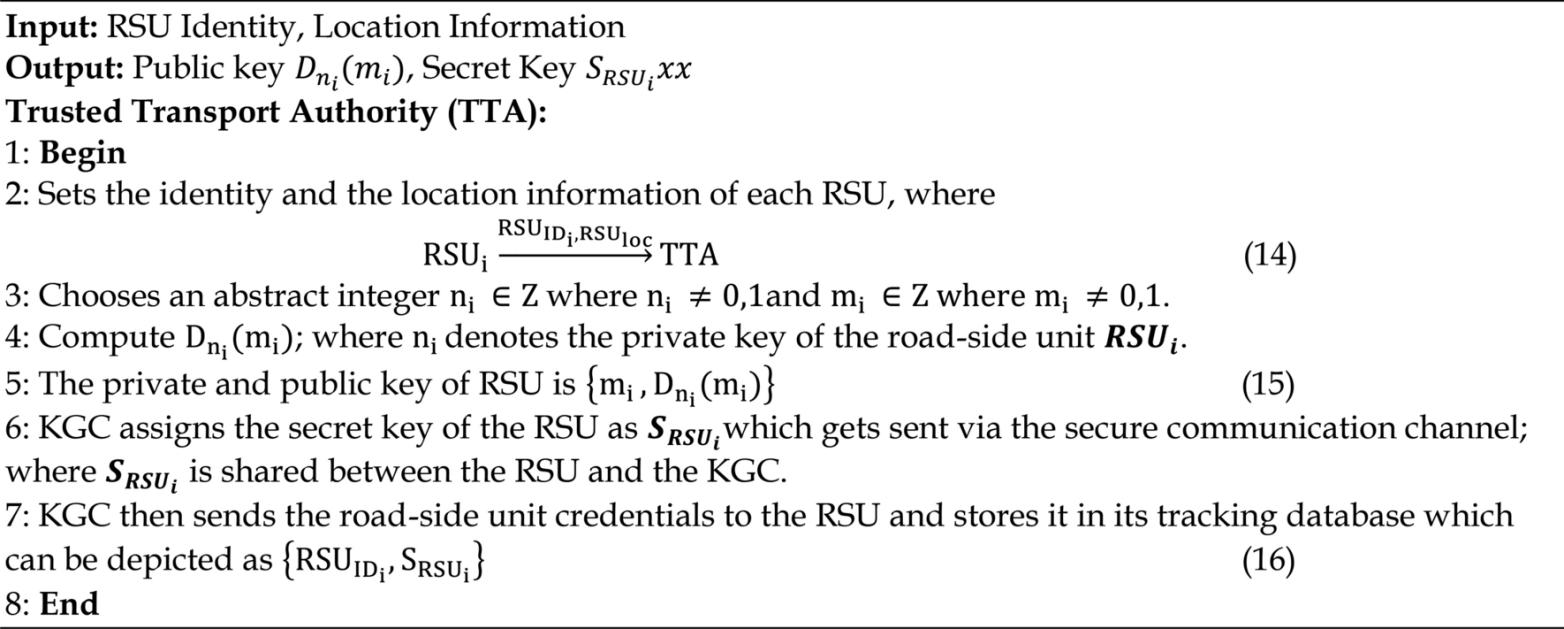
Offline RSU Registration Phase

### Dickson’s group key computation phase

**Fig. 3:**
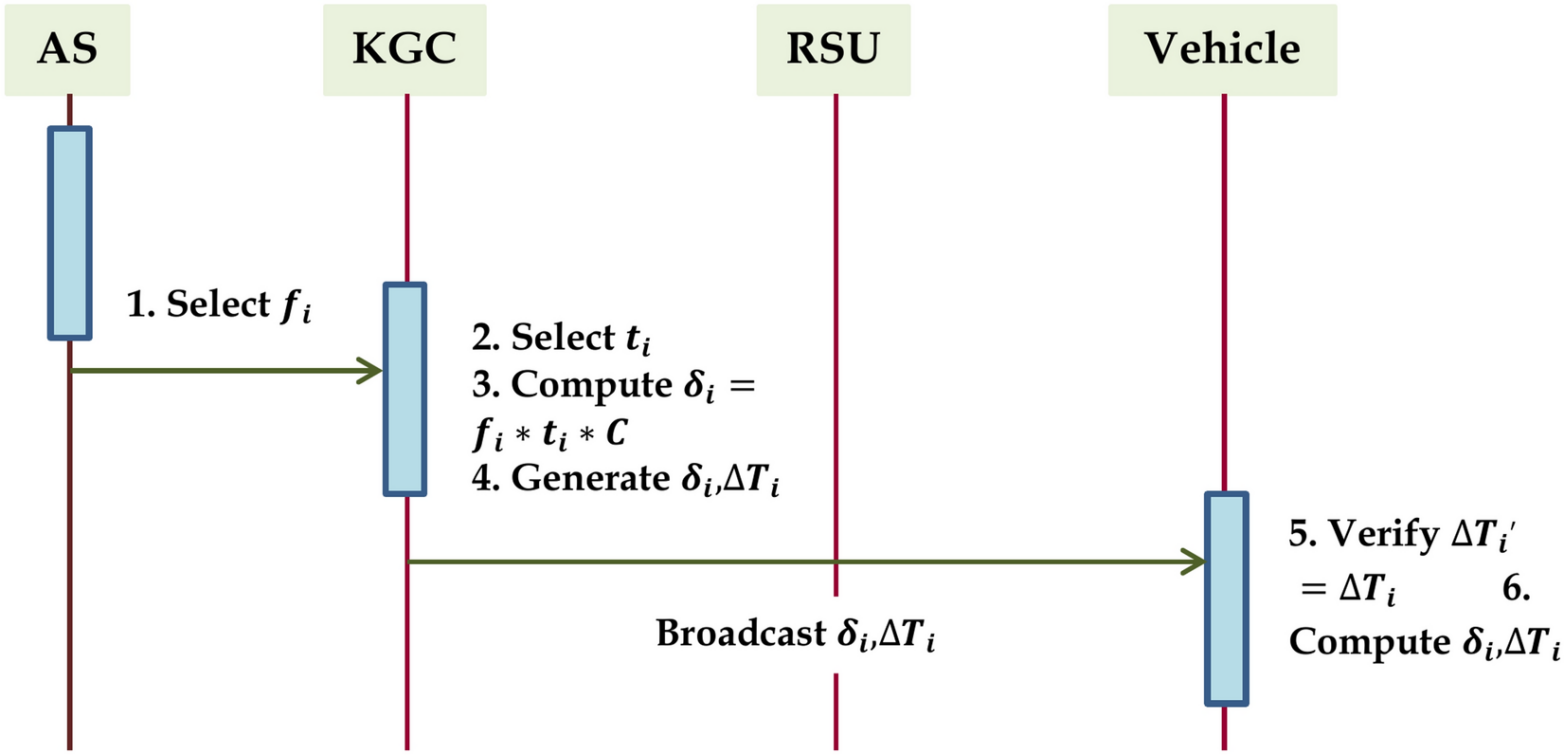
Group Key Computation and Distribution Phase.

Users or vehicles in the same communication RSU range must first go through an authorization process before they can receive a group key for message creation and authentication. A vehicle notifies the KGC of its approach to the RSU’s coverage region by sending an authentication request. The step 1 shows how the KGC and AS choose a new group key in response to the request. It then broadcasts the private key to all of the RSUs and cars in group G, as shown in step 2 and step 3 after calculating the private key for the vehicle that approaches the RSU coverage range. The step 4 shows how the vehicle can access a new domain key that was calculated using the CRT after it has been authorized. The list of steps needed to calculate the Dickson’s group key computation is given in Algorithm 4. The figure illustrates the group key computation and distribution phase in Fig. 3.

**Algorithm 4:**
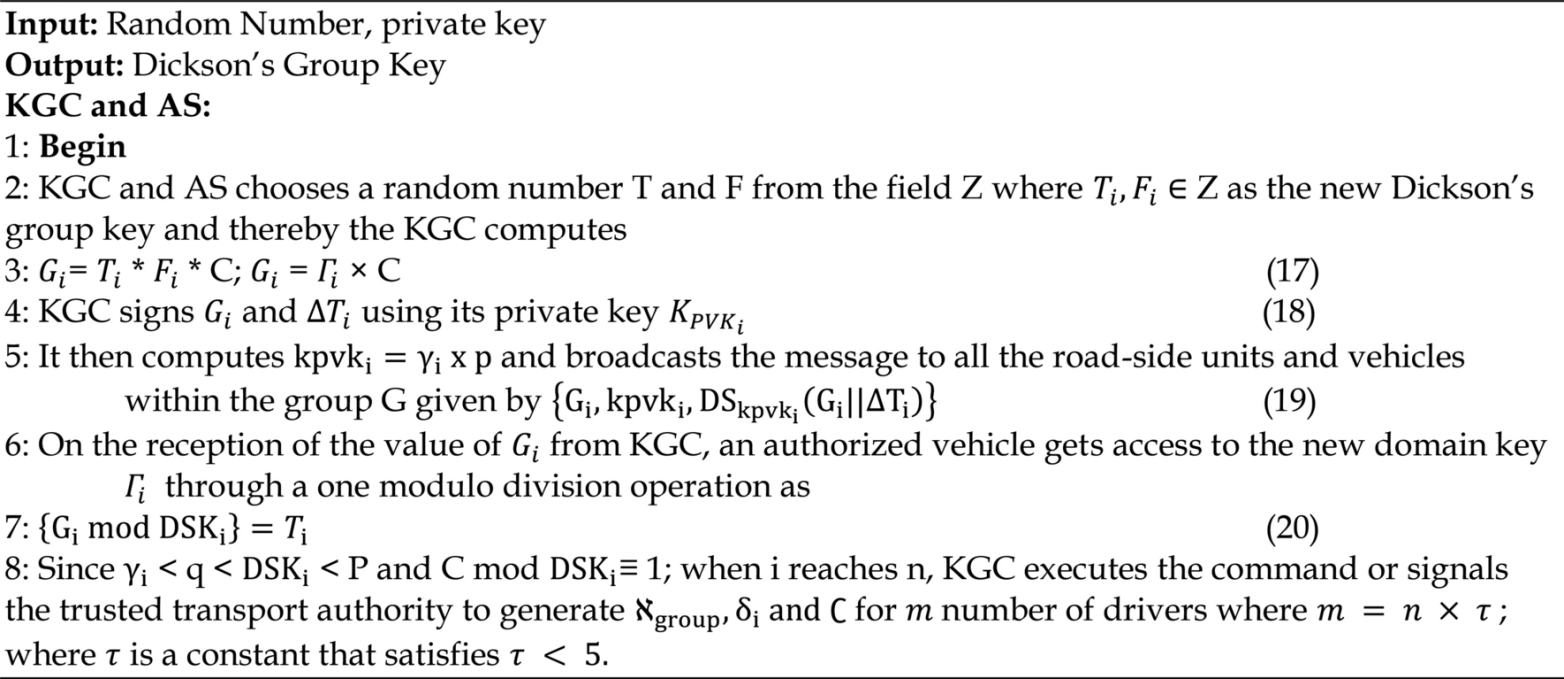
Dickson’s Group Key Computation Phase

### Group key distribution phase

In this phase, a pseudonym is given to every single vehicle before any communication is initiated. To produce the pseudonym for a vehicle, the Dickson polynomial must be calculated, as indicated using the step 1.Every vehicle communicates with the TTA using an authentication request message. Following receipt of the request, it directs the KGC to create a pseudo-identity allowing vehicle in order to enable communication, as indicated by the step 2. Every vehicle receives an authorized public key that may be used to describe the message using the step 3. Vehicles near an RSU need to be validated during this phase in order to ensure the group key’s authenticity. It comprises the generation of messages for authentication as well as their verification. Figure 5 describes how the vehicles are authenticated. Algorithm.5 has provided a description of the group key distribution. Prior to being sent from the car to the RSU, all messages are encrypted using the step 4, the Dickson polynomial, and the Dickson secret key.

**Algorithm 5:**
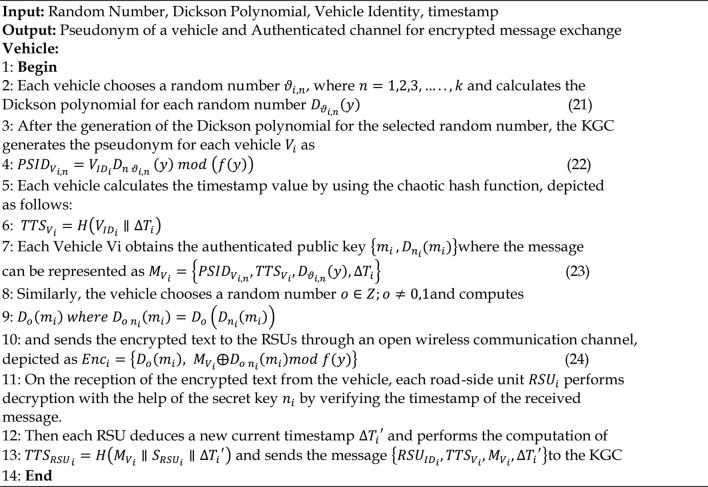
Group Key Distribution Phase

### Mutual authentication phase

**Fig. 4:**
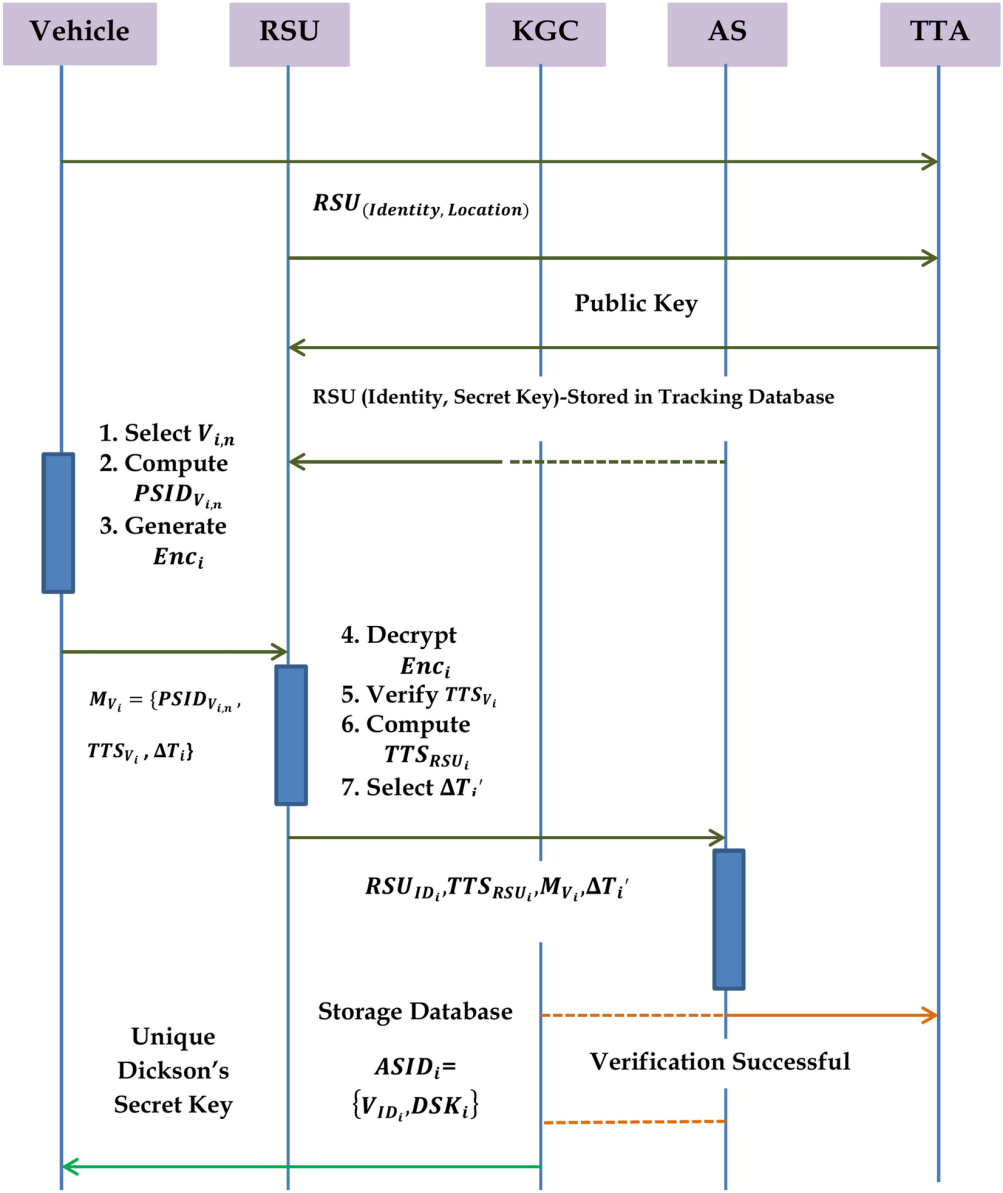
Authentication Protocol for a Vehicle.

Only after the AS computes the vehicle’s original identity using the matching Dickson polynomial and verifies the vehicles timestamp to get vehicle authorized. The original identification that has been decrypted is confirmed against the tracking database that is shared with the KGC and AS. Step 3 indicates that a vehicle is authentic if the matching vehicle identity is found in the database. Algorithm.6 has outlined the procedures that must be followed during the mutual authentication phase. Figure 4 provides the steps required for vehicle authentication.

**Algorithm 6:**
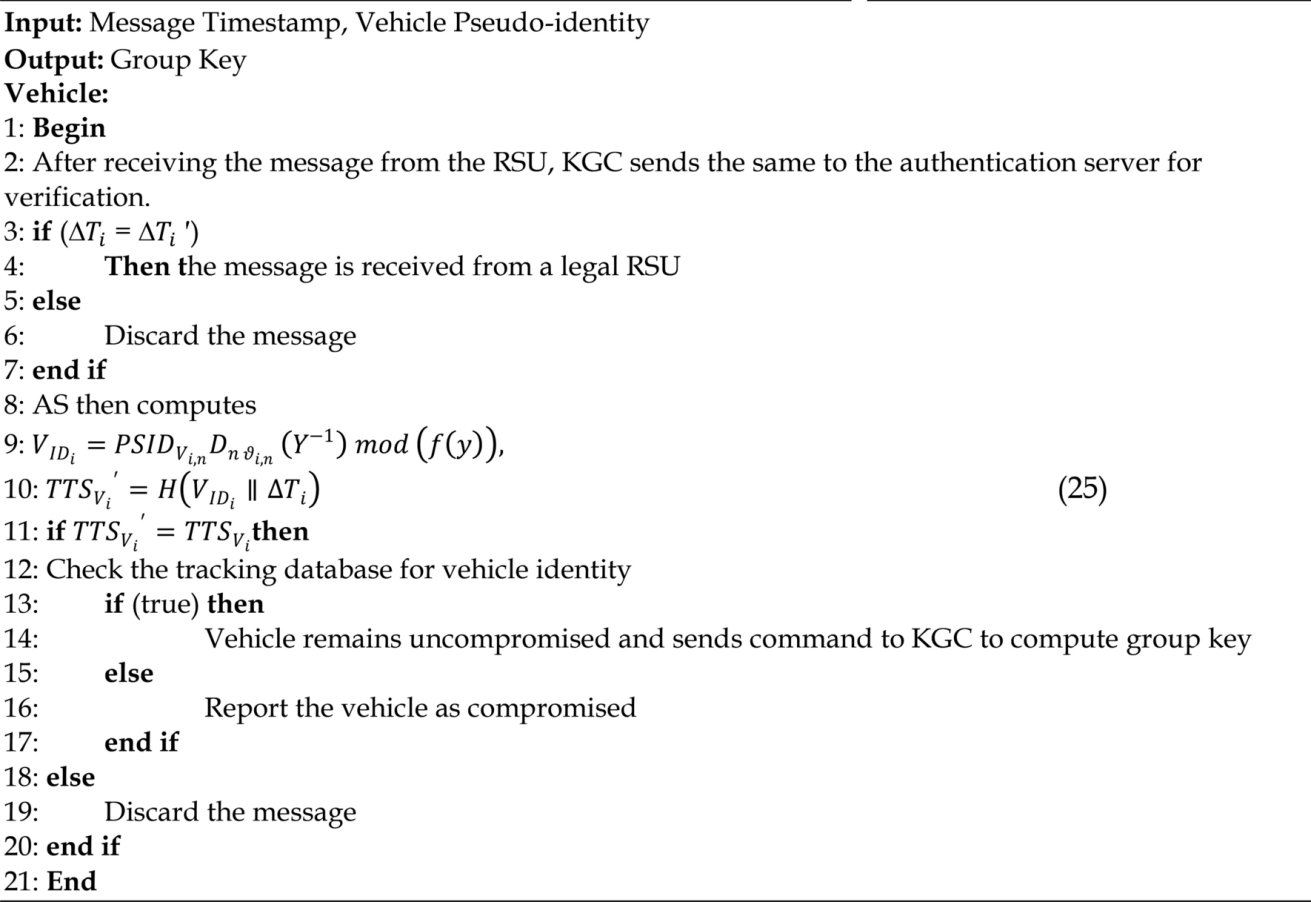
Mutual Authentication Phase

### Group key updation phase

The group key updating phase initiates when a vehicle leaves the RSU’s communication range, as shown by step 1 and step 4. Tree based logical key hierarchy rekeying procedure is followed where the group keys are updated dynamically^62^. The procedures to be followed during the group key updating phase are given in Algorithm 7.

**Algorithm 7:**
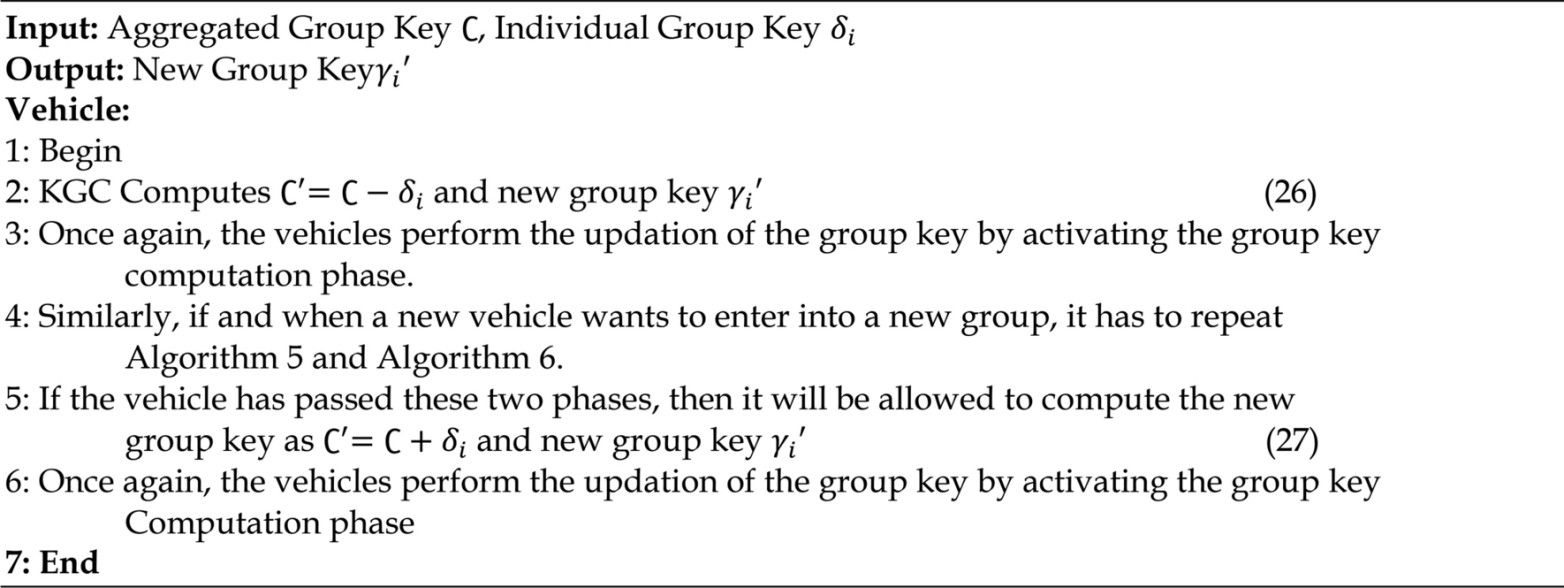
Group Key Updation Phase

### Message signature generation and verification phase

#### Message signature generation phase

According to Step 1 and 2, traffic information is transmitted by selecting a new pseudo-identity and a current timestamp, which is then sent to the vehicle or RSU for verification because the communication entities are authenticated using the Dickson secret key and the Dickson polynomial. The procedures to be followed during the message signing phase are given in Algorithm 8.

**Algorithm 8:**
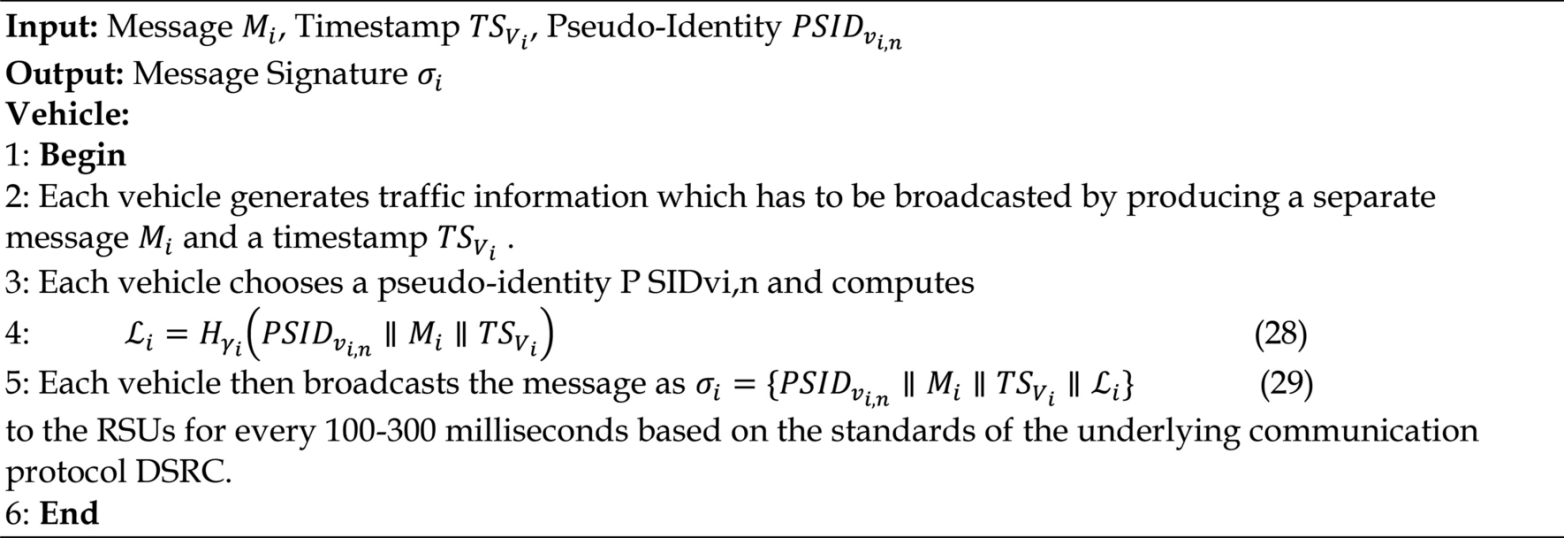
Message Signing Phase

#### Message signature verification phase

**Fig. 5:**
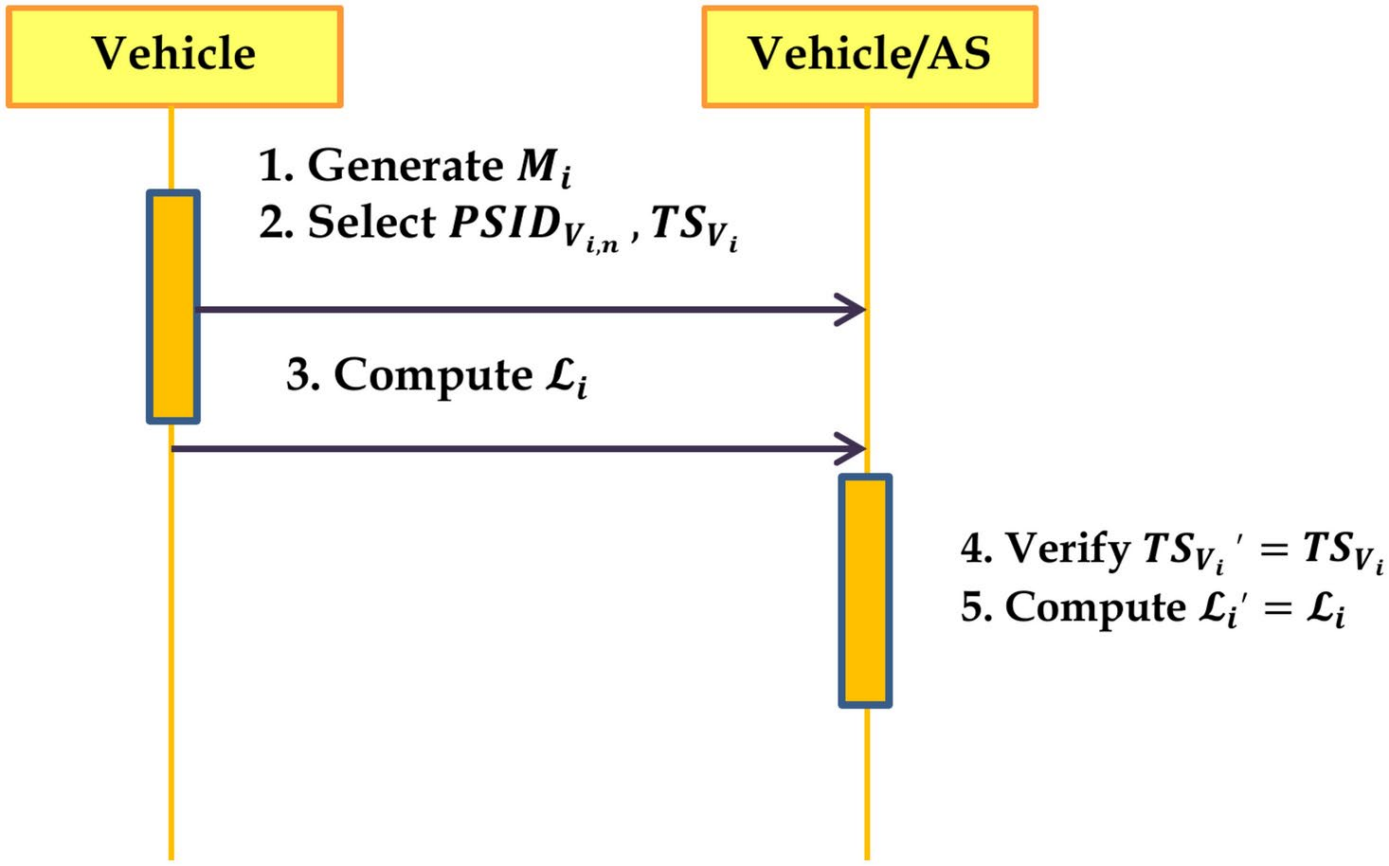
Message Authentication Phase.

When the message signature is received, the vehicle or the AS verifies it by utilizing step 1 and step 2 to compare the message signatures received. The step 2 is used to verify the correctness proof. The procedure for the single message verification is provided in Algorithm 9. Figure 5 provides the message authentication among the vehicles.

**Algorithm 9:**
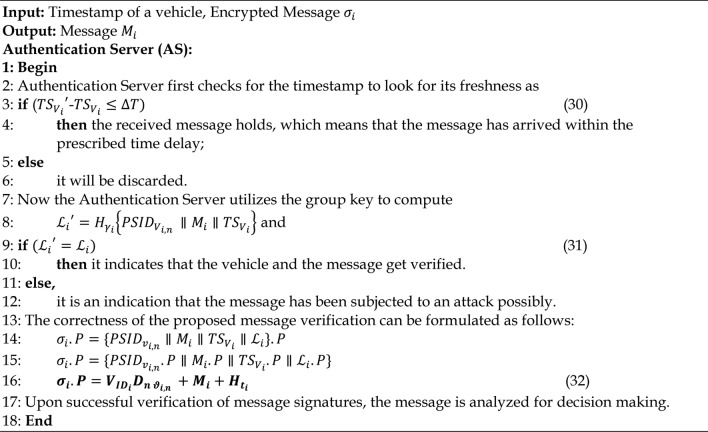
Single Message Verification

### Pseudonym updation phase

Every time the vehicle enters an urban region, it frequently changes its pseudonym to protect location privacy. Each vehicle produces a Dickson group polynomial from the pseudo-identity production phase, as indicated using the Eq. (33) as follows:

33 $$PSID_{{V_{i,n} }} = V_{{ID_{i} }} D_{{n \vartheta_{i,n} }} \left( y \right) mod \left( {f\left( y \right)} \right)$$

## Security analysis

This section provides the security analysis for the proposed authentication scheme in terms of formal security proofs and informal security proofs to validate its security strengths and its resilience to security attacks. Formal security analysis has been carried out by using BAN logic and ROR model. Since both of them serve for distinct purpose wherein BAN logic ascertain the logical correctness of the authentication proofs while ROR model verifies the security strengths of the authentication scheme.

### Formal security analysis based on BAN logic

This section provides an analysis based on BAN logic^63^ to validate the security features of the proposed distributed group key authentication scheme. The most important rules of the BAN logic are as follows:

- Axiom 1: Information-Definition Rule: $\frac{{R| \equiv R \leftrightarrow S, R \triangleleft Y_{k} }}{{R\left| { \equiv S} \right| \sim Y}}$
- Axiom 2: Time-Stamp Verification Rule: $\frac{{R| \equiv \# \left( Y \right), R\left| { \equiv S} \right| \sim Y}}{{R\left| { \equiv S} \right| \equiv Y}}$
- Axiom 3: Authority Rule: $\frac{{R\left| { \equiv S \Rightarrow Y, R} \right| \equiv S| \equiv Y}}{R| \equiv Y}$
- Axiom 4: Aliveness-Concurrence Rule: $\frac{R| \equiv \# \left( Y \right)}{{R| \equiv \# \left( {Y,Z} \right)}}$
- Axiom 5: Access Key Rule: $\frac{{R\left| { \equiv \# Y,R} \right| \equiv S| \equiv Y}}{{R| \equiv R\mathop \leftrightarrow \limits^{k} S}}$

There are four objectives which are supposed to be achieved and are as follows:

- **Obj 1:** $$TTA\left| { \equiv KGC} \right| \equiv RSU_{{ID_{i} }} ,~TTS_{{RSU_{i} }} ,~M_{{V_{i} }} ,~\Delta T_{i} ^{\prime }$$
- **Obj 2:** $$RSU_{i} | \equiv PSID_{{V_{i,n} }} , TTS_{{V_{i} }} ,D_{{\vartheta_{i,n} }} \left( y \right),\Delta T_{i}$$
- **Obj 3:** $$V_{i} | \equiv t_{i}$$
- **Obj 4:** $$V_{j} \left| { \equiv V_{i} } \right| \equiv PSID_{{v_{i,n} }} ,M_{i} ,TS_{{V_{i} }} ,{\mathcal{L}}_{i}$$

In order to carry out the formal security analysis, the messages are exchanged between the TTA, KGC, AS, RSUs and OBUs are formulated as follows:

- **Msg1:**$$V_{i} \to RSU_{j} :\left\{ {PSID_{{V_{i,n} }} , TTS_{{V_{i} }} ,D_{{\vartheta_{i,n} }} \left( y \right),\Delta T_{i} } \right\}$$
- **Msg2:** $$RSU_{j} \to KGC:\left\{ {RSU_{{ID_{i} }} ,~TTS_{{RSU_{i} }} ,~M_{{V_{i} }} ,~\Delta T_{i} ^{\prime } } \right\}$$
- **Msg3:** $$KGC \to V_{i} :g_{i}$$
- **Msg4:** $$V_{i} \to V_{j} :\left\{ {PSID_{{v_{i,n} }} ,M_{i} ,TS_{{V_{i} }} ,{\mathcal{L}}_{i} } \right\}$$

The rudimentary formulations required for the formal verification proofs are as follows:

- **P1:** $$RSU_{j} | \equiv RSU_{j} \mathop \leftrightarrow \limits^{{(m_{i } , D_{{n_{i} }} \left( {m_{i} } \right))}} V_{i}$$
- **P2:** $$RSU_{j} | \equiv \# \Delta T_{i}$$
- **P3:** $$RSU_{j} | \equiv V_{i} \Rightarrow PSID_{{V_{i,n} }} , TTS_{{V_{i} }} ,D_{{\vartheta_{i,n} }} \left( Y \right),\Delta T_{i}$$
- **P4:** $$KGC| \equiv \# \Delta T_{i}^{\prime }$$
- **P5:** $$KGC| \equiv RSU_{j} \Rightarrow \left( {RSU_{{ID_{i} }} ,S_{{RSU_{i} }} } \right)$$
- **P6:** $$V_{i} | \equiv V_{i} \mathop \leftrightarrow \limits^{{DSK_{i} }} TTA$$
- **P7:** $$V_{i} | \equiv \# \Delta T_{i}$$
- **P8:** $$V_{i} \left| { \equiv TTA} \right| \equiv DSK_{i}$$
- **P9:** $$V_{j} | \equiv V_{j} \mathop \leftrightarrow \limits^{{t_{i} }} V_{i}$$
- **P10:** $$V_{j} | \equiv \# TS_{{V_{i} }}$$

According to the formulated hypothesis and rational premises of BAN logic, the formal proof verification of the proposed authentication scheme is as follows:

By using the message 1, the following statements are obtained:

- **Q1:** $$RSU_{j} \triangleleft \left\{ {PSID_{{V_{i,n} }} , TTS_{{V_{i} }} ,D_{{\vartheta_{i,n} }} \left( y \right),\Delta T_{i} } \right\}_{{\left( {(m_{i } , D_{{n_{i} }} \left( {m_{i} } \right))} \right)}}$$

According to Q1, P1 and Axiom 1, it is obvious that:

- **Q2:** $$RSU_{j} \left| { \equiv V_{i} } \right| \sim PSID_{{V_{i,n} }} , TTS_{{V_{i} }} ,D_{{\vartheta_{i,n} }} \left( y \right),\Delta T_{i}$$

According to Q2, P2, Axiom 2 and Axiom 4, it is obvious that:

- **Q3:** $$RSU_{j} \left| { \equiv V_{i} } \right| \equiv PSID_{{V_{i,n} }} , TTS_{{V_{i} }} ,D_{{\vartheta_{i,n} }} \left( y \right),\Delta T_{i}$$.

According to Q3, P3 and Axiom 3, it is obvious that:

- **Q4:** $$RSU_{j} | \equiv PSID_{{V_{i,n} }} , TTS_{{V_{i} }} ,D_{{\vartheta_{i,n} }} \left( y \right),\Delta T_{i} \quad \left( {\text{Obj 2}} \right)$$

By using the message 2, the following statements are obtained:

- **Q5:** $$KGC\left| { \equiv RSU_{j} } \right| \sim RSU_{{ID_{i} }} ,~TTS_{{RSU_{i} }} ,~M_{{V_{i} }} ,~\Delta T_{i} ^{\prime }$$

According to Q5, P4, Axiom 2 and Axiom 4, it is obvious that:

- **Q6:** $$~KGC\left| { \equiv RSU_{j} } \right| \equiv RSU_{{ID_{i} }} ,~TTS_{{RSU_{i} }} ,~M_{{V_{i} }} ,~\Delta T_{i} ^{\prime }$$

According to Q6, P5 and Axiom 3, it is obvious that:

- **Q7:** $$KGC| \equiv RSU_{{ID_{i} }} ,~TTS_{{RSU_{i} }} ,~M_{{V_{i} }} ,~\Delta T_{i} ^{\prime } \quad \left( {{\text{Obj 1}}} \right)$$

By using the message 3, the following statements are obtained:

- **Q8:** $$V_{i} \triangleleft t_{i} x {\complement }$$

According to Q8, P6 and Axiom 1, it is obvious that:

- **Q9:** $$V_{i} \left| { \equiv KGC} \right|\sim t_{i} x {\complement }$$

According to Q9, P7, Axiom 2 and Axiom 4, it is obvious that:

- **Q10:** $$V_{i} \left| { \equiv KGC} \right| \equiv t_{i} x {\complement }$$

According to Q10, P8 and Axiom 3, it is obvious that:

- **Q11:** $$V_{i} | \equiv g_{i} \quad \left( {\text{Obj 3}} \right)$$

By using the message 4, the following statements are obtained:

- **Q12:** $$V_{j} \triangleleft PSID_{{v_{i,n} }} ,M_{i} ,TS_{{V_{i} }} ,{\mathcal{L}}_{i}$$

According to Q12, P9 and Axiom 1, it is obvious that:

- **Q13:** $$V_{j} \left| { \equiv V_{i} } \right| \sim PSID_{{v_{i,n} }} ,M_{i} ,TS_{{V_{i} }} ,{\mathcal{L}}_{i}$$

According to Q13, P10, Axiom 2 and Axiom 4, it is obvious that:

- **Q14:** $$V_{j} \left| { \equiv V_{i} } \right| \equiv PSID_{{v_{i,n} }} ,M_{i} ,TS_{{V_{i} }} ,{\mathcal{L}}_{i} \quad \left( {\text{Obj 4}} \right)$$

It is obvious that the proposed authentication scheme through the security analysis made using BAN logic has achieved all the formulated objectives and thereby provides an assurance of mutual authentication between the vehicles. It is also apparent that vehicles receive correct group key after the mutual authentication which makes vehicles to have a communication with the same group key within the range of road-side units. From these results obtained, it is obvious that our proposed authentication scheme according to the definition (1 and 2) it is not possible to crack due to the CMDDHP.

### Formal security analysis based on ROR model

In VANETs, the communication proceeds between the road-side units and the vehicles through a wide open wireless communication channel, it is obvious that the network is susceptible to hackers and malevolent users which are unavoidable. Analysis using ROR has been adopted from Vallant et al.^64^. To prove that our proposed authentication mechanism is safe from adaptive selected message attacks, the following proofs are presented.

#### Definition:

It is not possible for an invader to gain access to an entity $\left( {t, \beth , p} \right)$ to perform an adaptive chosen message attack under a signature authentication scheme. P denotes the number of $H_{2}$ hash queries in the random oracle.

#### Theorem 1:

The ROR states that any invader A with a probabilistic polynomial time executes the game (Definition 4) and succeeds with probability which cannot be left out in the corresponding polynomial time, then the simulator with the probabilistic polynomial time can solve CMDDHP problem not less than within a span of $\beth^{\prime} = \frac{\beth }{p}$ in that polynomial time.

#### Proof:

Let us assume that an invader $\chi$ can be able to gain access and generate the message as $\left\{ {PSID_{{v_{i,n} }} ,M_{i} ,TS_{{V_{i} }} ,{\mathcal{L}}_{i} } \right\}$. Let $\psi$ be an imposter that has been built on $\chi$, so $\psi$ describes the capability to provide solution to the CMDDHP problem executed by $\chi$ with a ruled out probability. With an instance sample $\left\{ {Z, q,y,f\left( y \right),p = q^{o} } \right\}$ of the CMDDHP problem, $\psi$ imposter oracles which are challenged by $\chi$ as follows:

*Setup*: The Imposter $\psi$ fix $p = q^{o}$ and chooses an abstract integer $\lambda_{i} \epsilon Z$ which is utilized to establish an anonymous set ${\text{PSID}}_{{v_{n} }} = \left\{ {PSID_{{v_{1} }} ,PSID_{{v_{2} }} , \ldots ,PSID_{{v_{n} }} } \right\}$ where $i \epsilon \left\{ {1,2, \ldots ,n} \right\}$.

34 $$PSID_{{v_{{i,n}} }} = \left\{ {\begin{array}{*{20}c} {{\text{PSID}}_{{vl_{{1,n}} }} = p;} & {iff~i = i^{*} } \\ {PSID_{{v_{{1,n}} }} = q^{o} ;} & {iff~i \ne i^{*} } \\ \end{array} } \right.$$

Imposter $\psi$ selects an abstract integer $t_{i} \epsilon Z$ and computes the public group key as $g_{i} = \gamma_{i} x {\complement }$. Then the imposter $\psi$ sends these parameters as $pms = \left\{ {Z, P,t_{i} {\complement }, H_{1} ,H_{2} } \right\}$ and the anonymous set $K_{{ID_{{i,n}} }}$ to the invader $\chi$.

*H*_*2*_* Hash Query*: If the invader $\chi$ makes an $H_{2}$ query by having a pseudo-identity $PSID_{{v_{i,n} }}$, the imposter $\psi$ examines whether the tuple $\left\langle {{\text{PSID}}_{{ID_{i} }} ,TS_{{V_{i} }} ,\tau _{{H_{2} }} } \right\rangle$ is present in the hash list $L_{{H_{2} }}$ or not. Iff the tuple presents in the list, then the imposter $\left\langle {\tau _{{H_{2} }} = H_{2} {\text{PSID}}_{{ID_{i} }} ,TS_{{V_{i} }} } \right\rangle$ dispatch to $\chi$. Else $\psi$ selects an abstract $\tau_{{H_{2} }} \in Z$ and then appends $\left\langle {{\text{PSID}}_{{ID_{i} }} ,TS_{{V_{i} }} ,\tau _{{H_{2} }} } \right\rangle$ it to the hash list $L_{{H_{2} }}$. At the end, $\psi$ gives $\left\langle {\tau _{{H_{2} }} = H_{2} {\text{PSID}}_{{ID_{i} }} ,TS_{{V_{i} }} } \right\rangle$ to $\chi$.

*H*_*3*_* Hash Query*: When an invader $\chi$ makes an $H_{3}$ query with the message $\left\langle {PSID_{{v_{i,n} }} ,M_{i} ,TS_{{V_{i} }} ,\tau_{{H_{2} }} } \right\rangle$, the imposter $\psi$ examines iff the tuple $\left\langle {PSID_{{v_{i,n} }} ,M_{i} ,TS_{{V_{i} }} } \right\rangle$ is already present in the list $L_{{H_{3} }}$ or not. Iff the tuple presents in the list, then the imposter $\tau _{{H_{3} }} = H_{3} \left\langle {{\text{PSID}}_{{ID_{i} }} ,TS_{{V_{i} }} ,M_{i} } \right\rangle$ dispatch to $\chi$. Else $\psi$ selects an abstract ${\tau }_{{H}_{3}}\in Z$ and then appends $\left\langle {{\text{PSID}}_{{ID_{i} }} ,TS_{{V_{i} }} ,M_{i} ,\tau _{{H_{2} }} } \right\rangle$ it to the hash list $L_{{H_{2} }}$. At the end, $\psi$ gives $\tau _{{H_{3} }} = H_{3} \left\langle {{\text{PSID}}_{{ID_{i} }} ,M_{i} ,TS_{{V_{i} }} } \right\rangle$ to $\chi$.

*Sign Query*: If the invader $\chi$ generates a signing inquiry on the message $M_{i}$ and the pseudo-identity ${\text{PSID}}_{{ID_{i} }}$, then the imposter $\psi$ examines the tuple value $\left\langle {{\text{PSID}}_{{ID_{i} }} ,TS_{{V_{i} }} ,\tau _{{H_{2} }} } \right\rangle$ from the hash list $L_{{H_{2} }} .$ Then the imposter $\psi$ picks up $\tau_{{H_{2} }}$ from the tuple $\left\langle {{\text{PSID}}_{{ID_{i} }} ,TS_{{V_{i} }} ,\tau _{{H_{2} }} } \right\rangle$.

If $i = i^{*}$; then the imposter $\psi$ selects three abstract integers $\phi_{i} ,\varphi_{i} ,{\Psi }_{i} \epsilon Z$, a random point ${\text{PSID}}_{{ID_{i} }}$; and computes $V_{{ID_{i} }} =$ $PSID_{{V_{i,n} }} D_{{n{ }\vartheta_{i,n} { }}} \left( {Y^{ - 1} } \right){ }mod{ }\left( {f\left( y \right)} \right)$. Then the imposter $\psi$ appends $\tau _{{H_{2} }} = H_{2} \left\langle {{\text{PSID}}_{{ID_{i} }} ,TS_{{V_{i} }} } \right\rangle ~and~\tau _{{H_{3} }} = \left\langle {H_{3} {\text{PSID}}_{{ID_{i} }} ,M_{i} ,TS_{{V_{i} }} } \right\rangle$ to the hash list $L_{{H_{3} }}$; then dispatches $\left\langle {{\text{PSID}}_{{ID_{i} }} ,TS_{{V_{i} }} ,M_{i} ,\tau _{{H_{2} }} } \right\rangle$ to $\chi$. According to the formulations specified, all the reply sent to the sign query becomes valid because $\left\langle {{\text{PSID}}_{{ID_{i} }} ,TS_{{V_{i} }} ,M_{i} ,\tau _{{H_{2} }} } \right\rangle$ has been answered in the game thereby satisfying:

35 $$\begin{gathered} \sigma_{i} \cdot P = V_{{ID_{i} }} D_{{n \vartheta_{i,n} }} + M_{i} + H_{{\gamma_{i} }} \hfill \\ = V_{{ID_{i} }} D_{{n \vartheta_{i,n} }} \left( Y \right)PSID_{{V_{i,n} }} D_{{n{ }\vartheta_{i,n} { }}} \left( {Y^{ - 1} } \right) + M_{i} + H_{{\gamma_{i} }} \hfill \\ = V_{{ID_{i} }} D_{{n \vartheta_{i,n} }} \left( Y \right)V_{{ID_{i} }} D_{{n{ }\vartheta_{i,n} { }}} \left( {Y^{ - 1} } \right) + M_{i} + H_{{\gamma_{i} }} \hfill \\ = PSID_{{ \vartheta_{i,n} }} \left( Y \right)V_{{ID_{i} }} D_{{n{ }\vartheta_{i,n} { }}} \left( {Y^{ - 1} } \right) + M_{i} + H_{{\gamma_{i} }} \hfill \\ = \sigma_{i}{\prime} \cdot P PSID_{{v_{i} }} D_{{n{ }\vartheta_{i,n} { }}} \left( {Y^{ - 1} } \right) \hfill \\ \sigma_{i} .P - \sigma_{i}{\prime} \cdot P = PSID_{{v_{i} }} D_{{n{ }\vartheta_{i,n} { }}} \left( {Y^{ - 1} } \right) \hfill \\ \end{gathered}$$

Else $i \ne i^{*}$; then the imposter $\psi$ gets verified that it has obtained a valid signature and publishes it.

*Output*: The invader communicates $\chi$ with the imposter $\psi$ until $\chi$ comprehend that the work has been completed. Therefore, the invader provides the message $\left\langle {{\text{PSID}}_{{ID_{i} }} ,TS_{{V_{i} }} ,M_{i} ,{\mathcal{L}}_{i} } \right\rangle$ as its output. The imposter checks whether the equation holds true or not.

36 $$\sigma_{i} .P = V_{{ID_{i} }} D_{{n \vartheta_{i,n} }} + M_{i} + H_{{\gamma_{i} }}$$

If not the imposter $\psi$ will end the process. By using the forgery axiom^54^, the invader may produce another legal valid message with $\left\langle {{\text{PSID}}_{{ID_{i} }} ,TS_{{V_{i} }} ,M_{i} ,{\mathcal{L}}_{i} ^{*} } \right\rangle$ within a polynomial time, if it selects another $H_{2}$ where $V_{{ID_{i} }} \ne V_{{ID_{i} }}^{*}$. Hence we obtain:

37 $$\sigma_{i}^{*} .P = V_{{ID_{i} }}^{*} D_{{n \vartheta_{i,n} }} + M_{i} + H_{{\gamma_{i} }}$$

From the Eqs. 36 and 37, it is deduced that

38 $$\begin{gathered} (\sigma_{i}^{*} - \sigma_{i} ) \cdot P = \sigma_{i} \cdot P - \sigma_{i}^{*} \cdot P \hfill \\ = (V_{{ID_{i} }} D_{{n \vartheta_{i,n} }} + M_{i} + H_{{\gamma_{i} }} ) - \left( {V_{{ID_{i} }}^{*} D_{{n \vartheta_{i,n} }} + M_{i} + H_{{\gamma_{i} }} } \right) \hfill \\ = P\left( {V_{{ID_{i} }} - V_{{ID_{i} }}^{*} } \right)\left( {D_{{n \vartheta_{i,n} }} + M_{i} + H_{{\gamma_{i} }} } \right) \hfill \\ \end{gathered}$$

Now the imposter $\psi$ produces the solution $\left( {V_{{ID_{i} }} - V_{{ID_{i} }}^{*} } \right)^{ - 1} (\sigma_{i}^{*} - \sigma_{i} )$ for the proposed instance of the CMDDHP problem. Else the imposition will be ceased.

The correct solutions for the proposed instance of the CMDDHP problem can be assured based on the co-occurrence of the events as follows:

- Event $E_{1}$: The invader $\chi$ produces and outputs a legal signature forgery.
- Event $E_{2}$: The invader $\chi$ can forfeit a pseudo-identity $PSID_{{v_{i} }} \ne PSID_{{v_{i} }}^{*}$

Due to Prob $\left[ {E_{1} } \right] = \beth$, Prob $\left[ {E_{2} } \right] = \frac{1}{p}$; we get.

Prob $\left[ {E_{1} E_{2} } \right]$ = Prob $\left[ {E_{1} } \right]$ Prob $\left[ {E_{2} } \right]$ = $\beth x \frac{1}{p} = \frac{\beth }{p}$.

It is obvious that the imposter $\psi$ can solve the instance of the CMDDHP problem with the advantage $adv_{\psi } = = \frac{\beth }{p}$. Thus, the imposter $\psi$ calculates y within a polynomial time with an waste probability with the advantage of $\frac{\beth }{p}$ namely the solution to the CMDDHP problem which implies that the theorem 1 can be satisfied. However it is difficult to solve the CMDDHP within a minimum span of time. Hence, it proved that our proposed authentication scheme is secure against forgery under the adaptive chosen message attacks.

### Formal security analysis using coq tool^65^

Coq is a formal security verification system that offers tools for the interactive creation of machine-checked proofs along with a programming language and logic for expressing mathematical prepositions, algorithms, and theorems. Coq has been extensively utilized in several fields, such as security verification for protocols based on polynomials cryptography, quantum communication^66^. Coq offers a high degree of assurance in the security of cryptographic protocols by enabling logical reasoning about their characteristics and behavior. The ability to formally reason about the behavior and characteristics of the protocol is one advantage of utilizing Coq for security verification of cryptographic protocols. This aids in locating possible weaknesses in the protocol and offers a means of demonstrating that it satisfies security standards.

### Informal security analysis

In order to evaluate the safety measures used in our suggested authentication technique, informal security analysis has been presented below.

#### Message integrity and authentication

Since the authentication scheme utilizes group key based authentication strategy; it is necessary for vehicle to be verified as legitimate if it wants to join a group and connect with others. The Hash value created by the sender can be affirmed by using the formula ${\mathcal{L}}_{i} = H_{{t_{i} }} \left( {PSID_{{v_{i,n} }} \left\| {M_{i} } \right\|TS_{{V_{i} }} } \right)$. In order to verify the integrity of the message received, the receiver verifies the legitimacy of the hash value generated by using the group key as ${\mathcal{L}}_{i} = = {\mathcal{L}}_{i}^{*}$. If it holds then the message will be accepted.

#### Perfect backward secrecy

An intruder can compromise a group’s security by intercepting the freshly produced group key $t_{i}$ and using it to unlock the private key $DSK_{i} ;$ of one of the group vehicles. A large collection of positive integers related to the multiplicative field Z is also required for the random number generator used to produce the private keys. This feature makes it so the enemy can’t break into any other cars’ safes. Since the adversary cannot access the communication that had place before their entry into the group, the proposed solution satisfies the backward secrecy condition.

#### Perfect forward secrecy

It is obvious that any invader cannot find out the current group key $t_{i}$ when leaving the group as formulated earlier in the backward secrecy technique. When a vehicle leaves its group, KGC removes it by subtraction which is the culmination of $y_{i}$ and $x_{i}$ and extracts $\delta_{i}$ from ${\complement }$ to produce ${\complement }^{\prime }$. The newly generated domain key value $g_{i}$ is formed by the culmination of the rekeying message ${\complement }^{\prime }$ and $\gamma_{i}^{\prime }$. Without any requirement of the vehicle’s secret key it is possibly to get access to the new group key being generated even after leaving the group. These left vehicles from the group with its key information can be able to gain access to the new group key $\gamma_{i}$, which is impractical to be sent as a broadcast message from the KGC. This means that the vehicle has to culminate its secret key with the integers from 1 to p, where p is the maximum group value. On a certain situation, the vehicle generates ${\mathfrak{H}} =$*ϰ*′ (i.e., $DSK_{i} x a = {\mathfrak{H}}$). After the reception of the $a$, a particular vehicle $V_{i}$ obtains a series of integers n, which will divide the number $a$. The set of integers can be defined as $\left\{ {a mod 1,a mod 2, \ldots .,a mod a} \right\} = = 0$ each represents the value of n. From this obtained sequence of integers, the integer $t_{i} \epsilon n$ is also involved in the generation of the new group key. Due to this fact, it is assumed that the size of $\gamma_{i}$ is a bits, then the invader has to perform the modulo operation of $2^{a}$. Hence, it is evident deducing the value of $\gamma_{i}$ by selecting a large $DSK_{i}$ value for a vehicle’s secret key is highly expensive and incurs high computational overhead. Let us assume that the size of $DSK_{i}$ is fixed as 1024 bits which was previously set to 128, 256 and 512 bits. The attacker (after leaving the domain) can conduct a subsequent brute force attack to acquire the new domain key by selecting exploitable values from the set n that divides the integer a. If this effort takes 1 $\mu$ s, then the total time taken will be $2^{n - 1} \mu$ s would elapse. Our suggested technique meets the forward secrecy requirement since an attacker cannot gain the domain key to access the present communication.

#### Conditional privacy preservation

*I. Identity Privacy Preservation:* In order to retrieve the original identity the invader has to compute $V_{{ID_{i} }} =$ $PSID_{{V_{i,n} }} D_{{n{ }\vartheta_{i,n} { }}} \left( {Y^{ - 1} } \right){ }mod{ }\left( {f\left( y \right)} \right)$. However, $\gamma_{i}$ is stored in the TTA, and $t$ being a random number the invader cannot be able to gain access to the original identity. Because, it not possible to compute the CDHP. Therefore, the adversary will not be able to learn the user’s true identity even if the pseudo identity $PSID_{{V_{i,n} }}$ is leaked. *ii. Location Privacy Preservation:* The proposed authentication scheme engulfs pseudonym updation mechanism, which means that the vehicles changes it pseudonyms constantly when it reaches the urban scenario thereby ensuring the location privacy.

#### Traceability

On any legal proceedings, KGC will trace back the original identity of the vehicle that has involved in any malfunction. It traces from its tracking database based upon the command from the trusted transport authority. Since KGC issues the pseudonyms required for the vehicle, it is impossible for any invader to guess the pseudo-identity since it was encrypted by using Dickson polynomial. KGC uses the original ID of the vehicle and the master secret n and computes $V_{{ID_{i} }} =$ $PSID_{{V_{i,n} }} D_{{n{ }\vartheta_{i,n} { }}} \left( {Y^{ - 1} } \right){ }mod{ }\left( {f\left( y \right)} \right)$. Our proposed authentication scheme uses Iris biometric for the vehicle identity verification, which provides an accurate way of tracing back the user’s identity by using its iris biometric. Iris biometric based recognition provides an efficient way to track back in case of any illegitimate signature sent by any vehicle.

#### Unlinkability

In our proposed authentication scheme, the message signature is generated by using a pseudo-identity $PSID_{{V_{i,n} }}$. Since each pseudo-identity utilized for generating the message signature is significantly distinct and the random number utilized to verify the identities is never reused. Therefore, no opponent could link multiple signatures from the same vehicle thereby ensuring unlinkability.

#### Anonymity

Utilization of pseudonym provides the protection of the vehicle’s original identity which makes difficult for an adversary to compute the original identity of the vehicle except TTA.

#### Mutual authentication

In the proposed protocol the authenticity of the vehicles and RSUs are verified by using Dickson polynomial by AS. AS first checks and verifies for the timestamp $\Delta T_{i}^{\prime }$ from the received message $\{ RSU_{{ID_{i} }} , TTS_{{V_{i} }} , M_{{V_{i} }} , \Delta T_{i}^{\prime } \}$ where $\Delta T_{i} = \Delta T_{i}^{\prime }$. If both are same, then the road-side unit is a legal identity which sends or relays the message. When the verification of the timestamp remains successful, then AS computes $V_{{ID_{i} }} =$ $PSID_{{V_{i,n} }} D_{{n{ }\vartheta_{i,n} { }}} \left( {Y^{ - 1} } \right){ }mod{ }\left( {f\left( y \right)} \right),{ }TTS_{{V_{i} }}^{\prime } = H\left( {V_{{ID_{i} }} \left\| {\Delta T_{i} } \right.} \right)$. Iff $TTS_{{V_{i} }}^{\prime } = TTS_{{V_{i} }}$, then it checks the identity of the vehicle in the tracking database which is shared between the trusted transport authority and the key generation center. f the vehicle ID is present in the database then it is an authenticated vehicle. It then sends the command verified to the KGC to compute the group key $DSK_{i}$. Hence mutual authentication is achieved.

#### Non-repudiation

The sender cannot be able to deny the message being sent since the message contains the pseudo-identity which is generated based on the Dickson polynomial. Since the original identity of the vehicle is encapsulated with the help of variable and timestamps, it is not possible for a vehicle user or RSU to deny the message being sent during vehicular communications. Thus our proposed scheme achieves Non-Repudiation.

## Resistance to attacks

### Impersonation attack

An invader willing to impersonate a vehicle to other vehicles or RSUs must have to generate a message $\left\{ {PSID_{{v_{i,n} }} ,M_{i} ,TS_{{V_{i} }} ,{\mathcal{L}}_{i}^{*} } \right\}$ thereby satisfying the equation $\sigma_{i} .P = V_{{ID_{i} }} D_{{n \vartheta_{i,n} }} + M_{i} + H_{{\gamma_{i} }}$. According to theorem 1, it is impossible for a polynomial adversary to copy a genuine message signature is self-evident. Hence, our proposed authentication scheme is able to resist impersonation attack.

#### Message modification attack

It is possible for an opponent to alter a message and then rebroadcast it. Since the messages are authenticated using the chaotic hash function H, it is challenging to produce a genuine hash without the group key $g_{i}$. When the message $\{ PSID_{{v_{i,n} }} ,M_{i} ,TS_{{V_{i} }} ,{\mathcal{L}}_{i}^{*} \}$ gets modified, it cause ${\mathcal{L}}_{i} \ne H_{{t_{i} }} \left( {PSID_{{v_{i,n} }} \left\| {M_{i} } \right\|TS_{{V_{i} }} } \right)$ which means that the message will not be verified and accepted. Hence, it is evident that our proposed scheme can be able to resist message modification attack.

#### Replay attack

Normally, a replay attack involves bad actors sending out the repeated copies of previous messages. In our proposed scheme, since timestamp is included in every message generated the recipients can verify the life of the timestamp of the authenticated messages by checking the condition $TS_{{V_{i} }}^{\prime } - TS_{{V_{i} }} \le \Delta T$ holds. Hence our proposed scheme is resilient towards replay attacks.

#### Coalition attack

It happen when several hackers or invaders collide together or attack together to get access to the private key. In the proposed authentication scheme, when the invaders attempts to compute the newly generated group key when the vehicles leave the group. Since it is obvious that the value of $\delta_{i}$ is subtracted from ${\complement }$, some of the vehicles cannot collide to get access to the newly generated group key $\gamma_{i}$ because the utilized pairwise relative prime number is expensively huge. Let us speculate that there are two invaders Invader $I_{1}$ has gain access to the key values $DSK_{1}$, $\gamma_{i}$ and the invader $I_{2}$ has gain access to the values $DSK_{3}$, $\gamma_{i}$ at time $\left( {t - 2} \right)$. When the time is $\left( {t - 1} \right)$, the invader probably may leave the group with its access privileges $DSK_{1} and$ $t_{i}$. When the time becomes $t$, the invader $I_{2}$ obtains the rekeying information $r_{g}$ from KGC and computes $\gamma_{i}$.When the time becomes $\left( {t + 1} \right)$, the invader $I_{2}$ left the group with its access privileges and both the invaders may exchange their access keys. However, it is not possible to gain access to the newly updated group key $\gamma_{i}$ broadcasted at time $\left( {t + 2} \right)$, because $\delta_{1}$ and $\delta_{3}$ are excluded from ${\complement }$. Hence, it is evident that our proposed authentication scheme can be able to resist coalition attacks.

#### MitM attack

Let’s pretend an enemy is lurking between any pair of cars, or RSUs. Since each communication happens by means of an authenticated group key which are directly involved by the Dickson polynomial, it is not possible to counterfeit the message signature which has been proved by using the theorem 1. Hence our proposed authentication scheme can be able to resist man-in-the-middle attacks.

#### Key exposure attack

There might be a possibility to gain access to the pseudo-identity while during the communication between any two entities involved. However it is not possible to guess the random number and the Dickson polynomial utilized to generate the pseudo-identity. Even if the key gets exposed, it is not possible for the invader to gain access to the original identity since it involves an expensive CDHP. Intruders cannot obtain or compute the distributed computed group key from both the KGC and AS without spending a lot of time and money. Therefore, our proposed method is secure against key-exposure attacks.

#### DoS attack

In the proposed authentication scheme follows the segregation of duties in producing the necessary keys, pseudo-identities, message signatures and verification which reduces the computation time when compared with its counterparts. In the proposed scheme, since we followed distributed approach for signature verification which has been employed to segregate to the AS following the process of verification. Hence the overhead at each component has been reduced which increases the computation speed to a greater extent. This implies that the entities present inside the network cannot deny its work even if it encounters multiple vehicles at the same time. Thus the proposed scheme is resilient to DoS attacks.

#### ESL attack

For each vehicle the pseudonym is generated by using the random number as defined as $PSID_{{V_{i,n} }} = V_{{ID_{i} }} D_{{n \vartheta_{i,n} }} \left( y \right) mod \left( {f\left( y \right)} \right)$. The timestamp value for each vehicle is computed by using the chaotic hash function as $TTS_{{V_{i} }} = H\left( {V_{{ID_{i} }} \left\| {\Delta T_{i} } \right.} \right)$. Each Vehicle $V_{i}$ obtains the authenticated public key $\left\{ {m_{i } , D_{{n_{i} }} \left( {m_{i} } \right)} \right\}$ where the message can be represented by the Eq. (23) as $M_{{V_{i} }} = \left\{ {PSID_{{V_{i,n} }} , TTS_{{V_{i} }} ,D_{{\vartheta_{i,n} }} \left( y \right),\Delta T_{i} } \right\}$. The group key of the vehicle gets computed by choosing a random number $o \in Z;o \ne 0,1$ and it computes $D_{o} \left( {m_{i} } \right) where D_{{o n_{i} }} \left( {m_{i} } \right) = D_{o} \left( {D_{{n_{i} }} \left( {m_{i} } \right)} \right)$ and sends the encrypted text to the RSUs through an open wireless communication channel which can be depicted as $Enc_{i} = \left\{ {D_{o} \left( {m_{i} } \right), M_{{V_{i} }} \oplus D_{{o n_{i} }} \left( {m_{i} } \right)mod f\left( y \right)} \right\}$ When the adversary tries to attack to retrieve the group key it is not possible to guess the random number. Additionally during the message generation the original identity is encapsulated with Dickson polynomial based on semi-group and irreducible property along with time stamp it not possible to compute $D_{{o n_{i} }} \left( {m_{i} } \right)$ due to CMDDHP. From the Eq. (23) the group key gets computed with a random value which hidden and random adding an additional layer of flexibility is making it more secure and adaptable for key generation and encryption. Hence the proposed scheme is resistant towards ESL attack.

#### Side-channel/TPD attacks

Existing authentication schemes attempts to load /install the master secret key into the TPD of the vehicle where no attacker can compromise it. However, this information can be hijacked by the adversary by means of side-channel attack. In our proposed scheme, since the pseudonym gets updated periodically (“Pseudonym Updation Phase” Section) using $PSID_{{V_{i,n} }} = V_{{ID_{i} }} D_{{n \vartheta_{i,n} }} \left( y \right) mod \left( {f\left( y \right)} \right)$. Thus our proposed scheme is resistant to Side-channel attacks.

### Comparison of security properties

This section provides a comparative analysis of the proposed authentication scheme in terms of the security characteristics to that of the existing CPPA-GKA schemes^23,25–27,29,30,35,37,40,42,48^. From the Table 4 conditional privacy is guaranteed by^23^^,^^25^^,^^35^^,^^40^^,^^42^^,^^48^, ours; while identity privacy and location privacy is guaranteed by two schemes^48^, ours; Key escrow freeness is ensured by^23^^,^^25^, ours. Authentication schemes which are resilient to ESL attacks are^23^^,^^25^^,^^35^, ours. The proposed scheme is resilient against coalition attack, DoS attack and key exposure attacks.

**Table 4:** Comparison of security parameters.

Properties	^23^	^25^	^26^	^27^	^28^	^35^	^40^	^42^	^48^	Ours
Message integrity	Yes	Yes	Yes	Yes	Yes	Yes	Yes	Yes	Yes	Yes
Mutual authentication	Yes	Yes	Yes	Yes	Yes	Yes	Yes	Yes	Yes	Yes
Group key agreement	No	No	No	No	No	No	Yes	Yes	No	Yes
Conditional privacy	Yes	Yes	No	No	No	Yes	Yes	Yes	Yes	Yes
Identity privacy	Yes	No	No	Yes	No	No	Yes	No	Yes	Yes
Location privacy	No	No	No	No	No	No	No	No	Yes	Yes
Traceability	Yes	No	Yes	No	Yes	No	Yes	No	Yes	Yes
Untraceability	No	Yes	No	No	No	No	No	No	No	No
Unlinkability	No	No	No	No	Yes	No	No	Yes	No	Yes
Anonymity	Yes	Yes	No	Yes	Yes	No	Yes	No	No	Yes
Confidentiality	Yes	Yes	No	Yes	Yes	Yes	Yes	Yes	Yes	Yes
Non-repudiation	No	No	No	No	Yes	No	No	No	No	Yes
Key escrow freeness	Yes	Yes	No	No	No	No	No	No	No	Yes
Forward secrecy	No	No	Yes	No	No	Yes	Yes	No	Yes	Yes
Backward secrecy	No	Yes	Yes	No	No	Yes	Yes	No	Yes	Yes
Replay attack	No	Yes	Yes	No	No	Yes	Yes	No	Yes	Yes
Impersonation attack	Yes	Yes	No	No	No	Yes	No	No	Yes	Yes
Modification attack	No	No	Yes	No	No	No	No	No	No	Yes
MiTM attack	Yes	No	Yes	No	No	No	Yes	No	No	Yes
Coalition Attack	No	No	No	No	No	No	No	Yes	No	Yes
Key exposure attack	No	No	No	No	No	No	No	No	No	Yes
DoS attack	No	No	No	No	No	No	No	No	No	Yes
ESL attack	Yes	Yes	No	No	No	Yes	No	No	No	Yes
Cloning attack	Yes	No	No	No	No	No	No	No	No	Yes
Physical/TPD/side-channel attack	Yes	No	No	No	Yes	No	No	No	No	Yes

## Experimental setup and performance metrics

The experimental setup of the proposed distributed GKA scheme has been carried out by using the NS3 and SUMO traffic simulator. To prototype the vehicular communications, OMNET++ must first expose and design the performance of vehicles in SUMO. The work has been implemented by using Ubuntu 18.04 LTS on ${\text{Intel\textregistered Core}}^{TM}$ i7- i7-10610U processor and 4.90 GHz and 8 GB RAM. The simulation parameters have been depicted by using the Table 5 as follows:

**Table 5:** Parameters of Experiment Simulation.

Specification	Configuration
Simulator	NS3
Duration of simulation	100–300 s
Communication range	50–300 m
Mobility model	Manhattan Grid
Area of simulation	3000*3000 $\left( {m^{2} } \right)$
Vehicle quantity	100
Speed	10 m/s
Momentum	8 m/s^2^
Power of transmission	40 mW
Data rate	24 Mbps
Size of a packet	215 Bytes

### Performance metrics

The performance aspects of the proposed distributed GKA scheme can be measured by using the performance metrics such as computation cost, communication cost and storage costs.

#### Computation cost (CMPC)

The work carried out on authentication scheme^32^ has employed the bilinear pairing operation which assumes a security parameter ${\text{\rm T}}$ of 180 bits which can be depicted as $\widehat{e}: G_{1 } x G_{1 } \to G_{T }$. In the proposed authentication scheme utilized Dickson polynomial based cryptography where $G_{1 }$ is an additive group of order $\hat{p}$ and the generator $\hat{q}$ which is a point on a on a multiplicative field Z where $E:D_{n} \left( x \right) \equiv \left( {2xD_{n - 1} \left( x \right) - D_{n - 2} \left( x \right)} \right) mod p;$ with an embedding degree of order 2. It is obvious to choose that the length of p be 160 solinas prime number and q be 512 bits. The authentication scheme utilized MIRACL cryptographic library software in order to perform the necessary cryptographic operation on a 64-bit Ubuntu 16.04 Operating system and an i4-Gen CPU. Table 6 provides the basic assumptions of various cryptographic operations. Table 7 summarizes the notations and run-time efficiency of various cryptographic operations has been adopted from^46,55,56^. Performance analysis has been evaluated by comparing the proposed authentication scheme along with the existing schemes such as^23,25,27,29,30^.

**Table 6:** Basic Group field assumptions of various cryptographic operations.

Method	Curve	Pairing	Cyclic Group	|P|	[G}	Group field range
Bilinear-pairing	$\overline{E}$: $y^{2}$ = $x^{3}$ + x mod $\hat{p}$	$\hat{e}$: $G_{1}$ x $G_{2}$ → $G_{T}$	$G_{1}$(P)	64 Bytes	q = 20 bytes	|$G_{1} | = 128$ bytes
Elliptic curve	$\overline{E}$: $y^{2} = x^{3} +$ ax + b mod p	No	G(P)	20 Bytes	q = 20 bytes	|G|= 40 Bytes
Chebyshev polynomial	$T_{n} \left( x \right) \equiv \left( {2xT_{n - 1} \left( x \right) - T_{n - 2} \left( x \right)} \right)\left( {mod P} \right);$ $n{ \succcurlyeq }2$	No	$Z_{p}^{*}$	–	–	$\left| {Z_{p}^{*} } \right| = 20$ Bytes
Dickson polynomial	$D_{n} \left( {x,\alpha } \right) = x D_{n - 1} \left( {x,\alpha } \right) - \alpha D_{n - 2} \left( {x,\alpha } \right);$ $n{ \succcurlyeq }2$	No	$Z_{p}^{*}$	–	–	$\left| {Z_{p}^{*} } \right| = 20$ Bytes

**Table 7:** Notations of different cryptographic operations.

Notations	Run-time (ms)	Output length in bits
$T_{emul}$	0.4420	256
$T_{eSign}$	1.536	256
$T_{eSym}$	0.087	256
$T_{everify}$	6.044	256
$T_{ea}$	0.2463	256
$T_{pm}$	2.5597	256
$T_{ddp}$	0.172	128
$T_{h}$	0.0045	256
$T_{C/d}$	0.0102	128
$T_{share}$	4.113	256
$T_{DH}$	0.00207	128
$T_{GHKEY}$	0.172	128
$T_{r}$	0.0106	256
$T_{f}$	0.0106	256
$T_{PR}$	0.0106	256
$T_{Puf}$	0.378	64
$T_{mod}$	3.0376	3072

The symbols utilized in the Table 4 are described as follows: $T_{emul}$ defines the run-time efficiency of elliptic curve multiplication operation; $T_{ea}$ defines the run-time efficiency of elliptic curve point addition operation; $T_{esm}$ defines the run-time efficiency of the elliptic curve scalar multiplication operation; $T_{ddp}$ defines the run-time efficiency of one scalar multiplication on Dickson polynomial; $T_{DH}$ defines the run-time efficiency of dickson encryption; $T_{h}$ defines the run-time efficiency of one hash function operation using secured chaotic hash algorithm; $T_{DH}$ defines the run-time efficiency of dickson encryption; $T_{GHKEY}$ defines the run-time efficiency of one keyed hash function operation chaotic maps; $T_{pm}$ denotes the run-time efficiency of point multiplication operation; $T_{pa}$ defines the run-time efficiency of point addition operation; $T_{C/d}$ defines the run-time efficiency of symmetric encryption/decryption; $T_{r}$ define the run-time efficiency of random number generation; $T_{f}$ defines the run-time efficiency of fuzzy extractor; and $T_{PR}$ defines the run-time efficiency of the pseudo-random function.

The total computation cost can be calculated by using the formula given in the Eq. (39).

39 $$\begin{aligned} Computation Cost \left( {CMPC} \right) & = Parameter + Group Key computation \\ & \quad + distribution + Mutual Authentication + Pseudonym updation \\ & \quad + Group key updation + Message Signing Cost + Verification Cost \\ \end{aligned}$$

#### Communication cost (CMMC)

For the computation of the communication cost, it is assumed that $G_{1}$ and $G$ are 128 bytes and 40 bytes. In case of addition the result of the hash function operation and the size of the timestamp can be calculated as 20 bytes and 4 bytes. It is also identified since our scheme utilizes finite field $Z_{p}^{*}$ which involves the message size of 20 bytes. Communication cost can be calculated by using the Eq. (40) which is depicted as follows:

40 $$Communication Cost \left( {CMMC} \right) = \mathop \sum \limits_{i = 1}^{n} \begin{array}{*{20}c} {Group Element Length*} \\ {number of operation \left( n \right) + 2^{o} } \\ \end{array}$$

#### Storage cost (SC)

Storage cost can be defined as the number of bits required to perform authentication where the parameters are necessary to be stored. Storage cost is evaluated in terms of bits. The formula to compute the storage cost has been defined using the Eq. (41) as follows:

41 $$Storage Cost \left( {SC} \right) = \mathop \sum \limits_{i = 1}^{n entities} No of bits$$

## Results discussion and analysis

The distributed group key authentication scheme’s performance is analyzed here, taking into account factors like computation cost. The performance evaluation was completed using the aforementioned techniques as references depicted in Table 8. A comparative analysis of existing systems using characteristics such as fundamental hard problem, nature of security, grouping type, and presence/absence of bilinear pairing procedures has been provided in the table.

**Table 8:** Performance comparison based on various aspects of existing authentication schemes.

Schemes	Problem	Bilinear pairing/non-bilinear pairing/polynomial	Vulnerability/resistant
Liang et al.^23^	DLP, CDHP	Elliptic Curve Cryptography	Vulnerable
Kumar et al.^25^	DLP	Elliptic Curve Cryptography	Vulnerable
Zhang et al.^27^	In-distinguishability, Collision-Resistant	Threshold Sharing Scheme	Vulnerable
Roy et al.^29^	DLP	Elliptic Curve Cryptography	Resistant
Kumar et al.^30^	CMCDHP	Secret Sharing Scheme	Vulnerable
Proposed	CMDDHP, Irreducibility	Dickson Polynomial	Resistant

### Computation cost

**Fig. 6:**
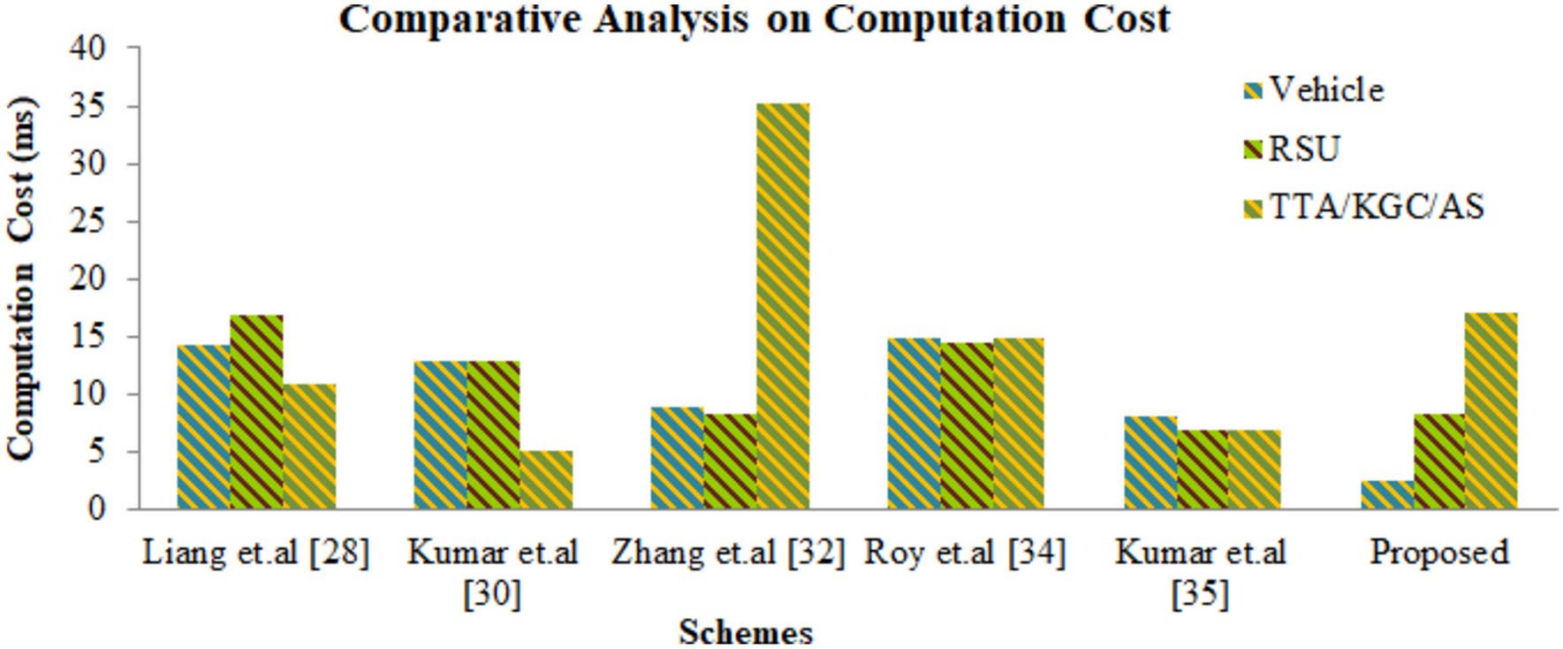
Comparison graph for Computation cost.

The cost of computation can be defined as the cost involved in performing all the cryptographic operations by the communicating entities inside the network. The proposed authentication scheme involves the entities such as vehicle, RSUs and TTA/KGC/AS. Therefore the computation cost incurred by the proposed authentication scheme with the vehicle can be defined as 4*T*_*DH*_ + 2*T*_*h*_ + *T*_*r*_ + *T*_*mul*_ + *T*_GHKEY_ + *T*_*key*_ + *T*_*sign*_ = 4 * 0.00207 + 2 * 0.0045 + 0.0106 + 0.2463 + 0.172 + 0.4420 = 0.00828 + 0090 + 0.0106 + 0.2463 + 0.172+ 0.4420 + 1.536 = 2.42418 ms. Similarly, the computation cost pertaining to RSUs is computed as 4*T*_*ddp*_  + 4*T*_*DH*_ + 2*T*_*h*_ + *T*_*GHKEY*_ + *T*_*sign*_ + *T*_*verify*_ =  4 * 0.172 + 4 * 0.00207 + 2 * 0.0045 + 0.172 + 1.536 + 6.044 = 8.28114  ms. Similarly in case of TTA/KGC/AS the computation cost can be defined as 6*T*_*r*_ + 3*T*_*GHKEY*_ + 4*T*_*key*_ + *T*_*ddp*_ + 2*T*_*DH*_ + 5*T*_*h*_ + T_*verify*_ = 6 * 0.0106 + 3 * 0.172 + 4 * 2 .5597 + 2 * 0.00207 + 0.172 + 5 * 0.0045 + 6.044 = 0.0636 + 0.516 + 10.2388 + 0.00414 + 0.172 + 0.0225 + 6.044 = 17.0569 ms. Therefor the total computation cost incurred by the proposed protocol = 17.2474 ms. In a similar manner, for other existing schemes^23,25,27,29,30^, the computation cost is estimated and a comparative analysis has been presented using the Table 9 and Fig. 6. Table 9 provides the performance comparison in terms of computation costs.

**Table 9:** Comparison on Computation Cost.

Schemes	$V_{{ID_{i} }}$	RSU	TTA/KGC/AS	Total
^23^	$$\begin{gathered} 5T_{{pm}} + T_{{ea}} + 3T_{{puf}} \hfill \\ + 8T_{h} = {\text{14}}.{\text{2148}}~{\text{ms}} \hfill \\ \end{gathered}$$	$$\begin{gathered} 6T_{{pm}} + T_{{ea}} + 3T_{{puf}} \hfill \\ + 9T_{h} = {\text{16}}.{\text{779}}~{\text{ms}} \hfill \\ \end{gathered}$$	$$\begin{gathered} 4T_{r} + 2T_{{ea}} + 4T_{{mul}} \hfill \\ + 4T_{h} = {\text{1}}0.{\text{7918}}~{\text{ms}} \hfill \\ \end{gathered}$$	$$\begin{gathered} 15T_{{pm}} + 4T_{{ea}} + 6T_{{puf}} \hfill \\ + 21T_{h} = {\text{41}}.{\text{7856}}~{\text{ms}} \hfill \\ \end{gathered}$$
^25^	$$\begin{gathered} 8T_{h} + 5T_{{pm}} + T_{{C/d}} \hfill \\ + T_{r} = {\text{12}}.{\text{8643}} \hfill \\ \end{gathered}$$	$$\begin{gathered} 7T_{h} + 5T_{{pm}} + 2T_{{C/d}} \hfill \\ + 2T_{r} = {\text{12}}.{\text{87}}0{\text{2}} \hfill \\ \end{gathered}$$	$$\begin{gathered} 5T_{h} + 2T_{{pm}} + 3T_{{C/d}} \hfill \\ + T_{r} = {\text{5}}.{\text{1821}} \hfill \\ \end{gathered}$$	$$\begin{gathered} 20T_{h} + 12T_{{pm}} + 6T_{{C/d}} \hfill \\ + 5T_{r} = {\text{3}}0.{\text{9166}} \hfill \\ \end{gathered}$$
^27^	$$\begin{gathered} T_{{pm}} + 2T_{h} + 2T_{r} \hfill \\ + T_{{verify}} = {\text{8}}.{\text{9477}}~{\text{ms}} \hfill \\ \end{gathered}$$	$$\begin{gathered} 3T_{h} + 2T_{{pm}} + 3T_{{pa}} + T_{{sign}} \hfill \\ + T_{{verify}} = {\text{8}}.{\text{295}}~{\text{ms}} \hfill \\ \end{gathered}$$	$$\begin{gathered} 2T_{h} + 2T_{{pm}} + 2T_{r} + 3T_{{pa}} \hfill \\ + T_{{share}} + T_{{dec}} \hfill \\ = {\text{35}}.{\text{1426}}~{\text{ms}} \hfill \\ \end{gathered}$$	$$\begin{gathered} 6T_{h} + 4T_{{pm}} + 3T_{{pa}} + T_{r} \hfill \\ + T_{{share}} + T_{{verify}} \hfill \\ = {\text{19}}.{\text{8914}}~{\text{ms}} \hfill \\ \end{gathered}$$
^29^	$$\begin{gathered} 11T_{h} + 2T_{{C/d}} + 4T_{r} \hfill \\ + 2T_{{pm}} + T_{{mod}} \hfill \\ = {\text{14}}.{\text{9223}}~{\text{ms}} \hfill \\ \end{gathered}$$	$$\begin{gathered} 9T_{h} + 2T_{{C/d}} + 3T_{r} \hfill \\ + 2T_{{pm}} + T_{{mod}} \hfill \\ = {\text{14}}.{\text{3654}}~{\text{ms}} \hfill \\ \end{gathered}$$	$$\begin{gathered} 15T_{h} + 6T_{{C/d}} + 2T_{r} \hfill \\ + 2T_{{pm}} + T_{{mod}} \hfill \\ = {\text{14}}.{\text{9117}}~{\text{ms}} \hfill \\ \end{gathered}$$	$$\begin{gathered} 35T_{h} + 10T_{{C/d}} + 6T_{r} \hfill \\ + 6T_{{pm}} + 3T_{{mod}} \hfill \\ = {\text{44}}.{\text{1994}}~{\text{ms}} \hfill \\ \end{gathered}$$
^30^	$$\begin{gathered} T_{{sign}} + T_{{verify}} + 2T_{r} \hfill \\ + T_{{mul}} = {\text{8}}.0{\text{342}}~{\text{ms}} \hfill \\ \end{gathered}$$	$$\begin{gathered} 4T_{{ea}} + 5T_{h} + T_{{sign}} \hfill \\ + T_{{verify}} + T_{{sym}} \hfill \\ + T_{{share}} = {\text{6}}.{\text{83}}0{\text{7}}~{\text{ms}} \hfill \\ \end{gathered}$$	$$\begin{gathered} 4T_{{ea}} + 5T_{h} + T_{{sign}} \hfill \\ + T_{{verify}} + T_{{sym}} \hfill \\ + T_{{share}} = {\text{6}}.{\text{83}}0{\text{7}}~{\text{ms}} \hfill \\ \end{gathered}$$	$$\begin{gathered} 8T_{{ea}} + 10T_{h} + 3T_{{sign}} \hfill \\ + 3T_{{verify}} + 2T_{{sym}} \hfill \\ + 2T_{{share}} = {\text{21}}.{\text{6956}}~{\text{ms}} \hfill \\ \end{gathered}$$
Proposed	$$\begin{gathered} 4T_{{DH}} + 2T_{h} + T_{r} \hfill \\ + T_{{mul}} + T_{{GHKEY}} \hfill \\ + T_{{key}} = {\text{2}}.{\text{42418}}~{\text{ms}} \hfill \\ \end{gathered}$$	$$\begin{gathered} 4T_{{ddp}} + 4T_{{DH}} + 2T_{h} \hfill \\ + T_{{GHKEY}} + T_{{sign}} \hfill \\ + T_{{verify}} = 8.28114 \hfill \\ \end{gathered}$$	$$\begin{gathered} 6T_{r} + 3T_{{GHKEY}} + 4T_{{key}} \hfill \\ + T_{{ddp}} + 2T_{{DH}} + 5T_{h} \hfill \\ + T_{{verify}} = {\text{17}}.0{\text{569}}~{\text{ms}} \hfill \\ \end{gathered}$$	$$\begin{gathered} 7T_{r} + 5T_{{GHKEY}} + 4T_{{key}} + 5T_{{ddp}} \hfill \\ + 10T_{{DH}} + 9T_{h} + T_{{sign}} \hfill \\ + 2T_{{verify}} = {\text{17}}.{\text{2474}}~{\text{ms}} \hfill \\ \end{gathered}$$

From the Table 9 it is obvious that the proposed authentication scheme incurs lower computation overhead at the vehicle side and RSUs while at TTA/KGC/AS the computation cost is bit higher. However, the overall computational cost for the entire message transmission is very less due to the use of less computation based on Dickson polynomial whose security strength is extremely good than the existing counterparts. Since distributed key generation based mutual authentication is performed the proposed work incurs less computation costs.

### Communication cost

**Fig. 7:**
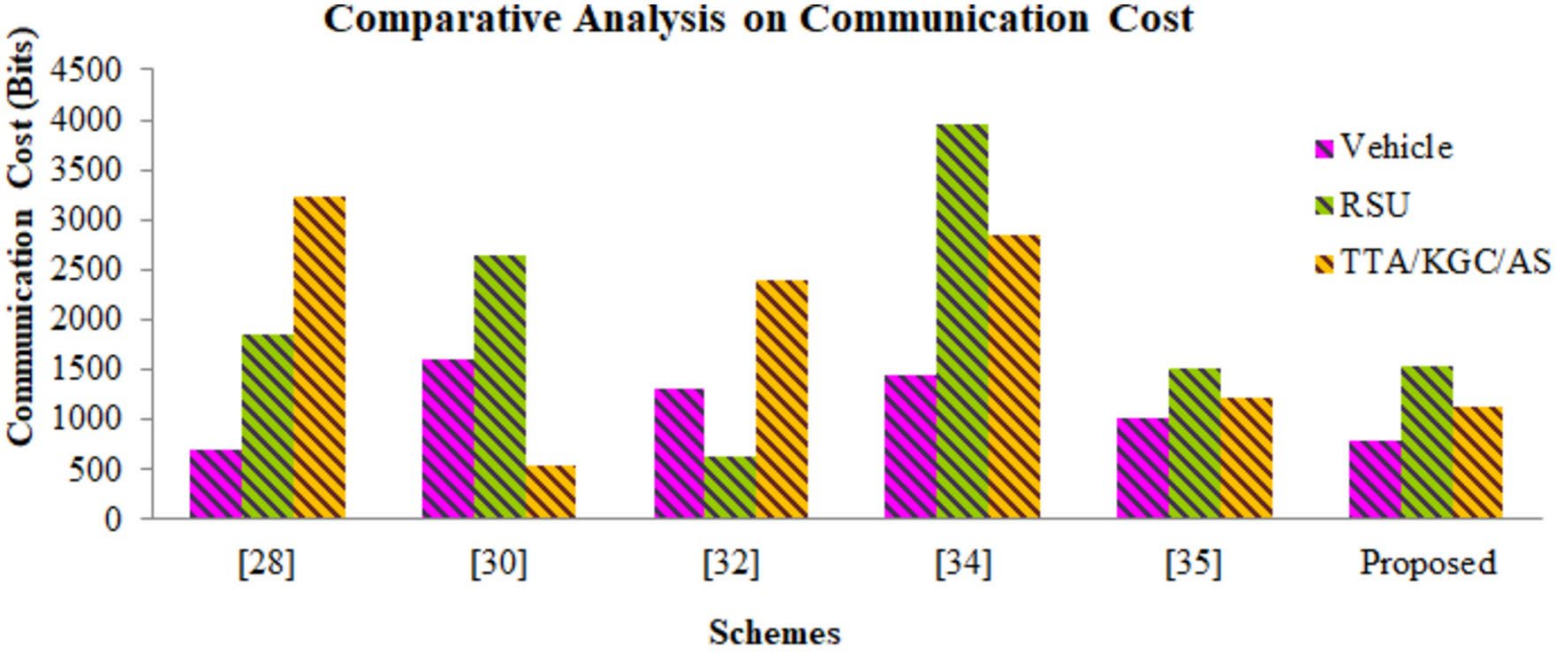
Comparison graph for communication cost.

Figure 7 provides the comparative analysis on the communication cost for the proposed authentication scheme to that of the existing schemes. Table 10 provides the computation and the comparison of the communication cost. The authentication delay is greatly reduced due to the use of chaotic hash function of 256 bits. The proposed Dickson polynomial provides an encrypted message length of 3456 bits. The communication cost for the proposed protocol at the vehicle side can be computed as $M_{{V_{i} }} = \left\{ {PSID_{{V_{i,n} }} , TTS_{{V_{i} }} ,D_{{\vartheta_{i,n} }} \left( y \right),\Delta T_{i} } \right\}$ and $D_{{o n_{i} }} \left( {m_{i} } \right) = D_{o} \left( {D_{{n_{i} }} \left( {m_{i} } \right)} \right)$ is defined as 800 bits. While at RSUs, the communication cost can be computed as ${\mathcal{L}}_{i}^{\prime } = H_{{\gamma_{i} }} \left\{ {PSID_{{V_{i,n} }} \left\| {M_{i} } \right\|TS_{{V_{i} }} } \right\}$ and $\sigma_{i} .P = \left\{ {PSID_{{v_{i,n} }} \left\| {M_{i} } \right\|\left. {TS_{{V_{i} }} } \right\|{\mathcal{L}}_{i} } \right\} \cdot P$ which involves 1536 bits and at the TTA/KGC/AS side the cost of communication is 1120 bits. The overall cost of communication for a message transmission can be 3456 bits. The authentication scheme proposed incurs lower communication cost when compared with that of its counterparts. In a similar way, the communication cost for the other existing schemes^23,25,27,29,30^ are computed and compared which is depicted in the Table 10 and Fig. 7.

**Table 10:** Comparison on Communication Cost.

Schemes	$V_{{ID_{i} }}$	RSU	TTA/KGC/AS	Total
^23^	704	1856	3230	5790
^25^	1600	2656	544	4800
^27^	1312	640	2400	4352
^29^	1440	3968	2848	7872
^30^	1020	1504	1216	3740
Proposed	800	1536	1120	3456

### Storage cost

**Fig. 8:**
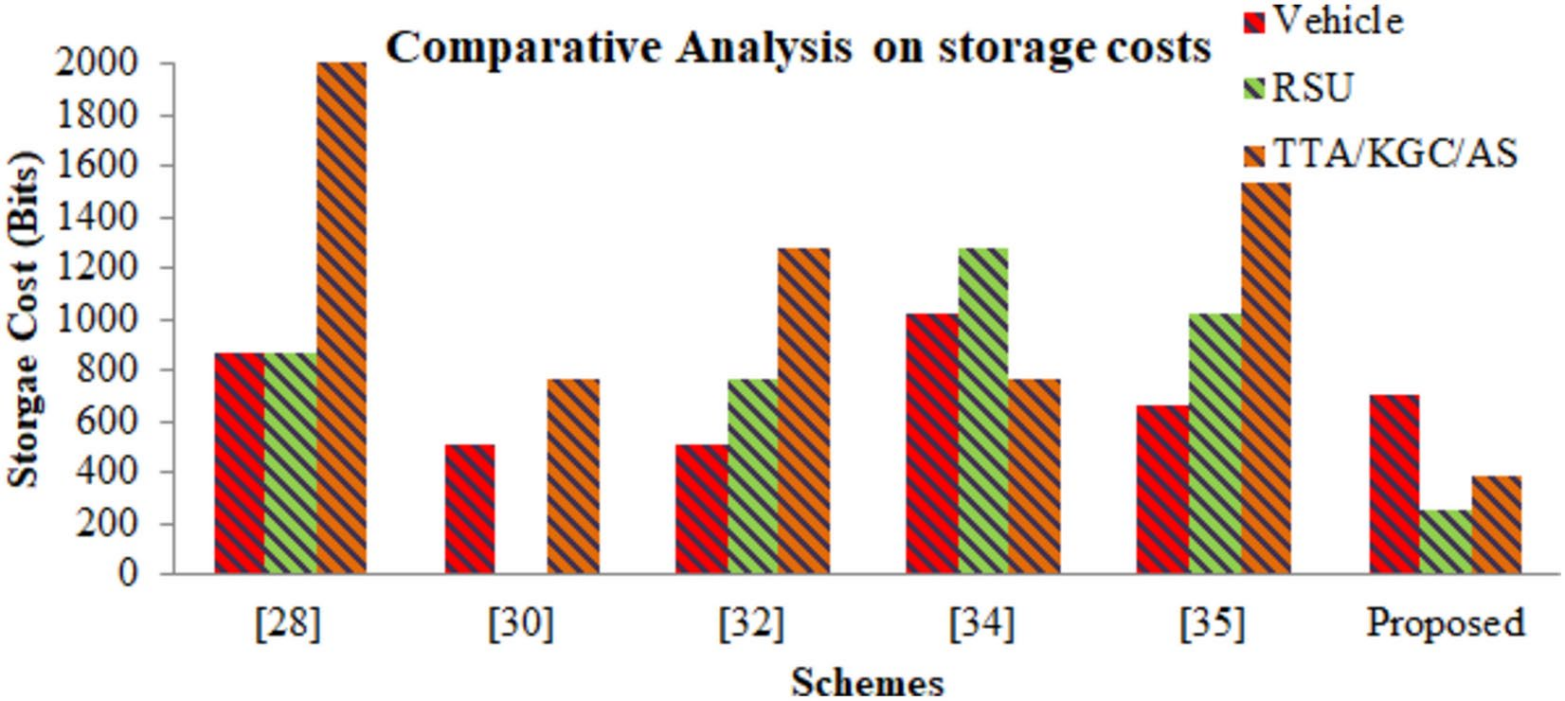
Comparison graph for storage overhead.

The overall storage cost can be computed by the number of bits needed by the entities inside the network. It is presumed that the cost of system parameters initialized during the system setup and vehicle registration are considered to be zero. The proposed authentication scheme involves the storage cost at the vehicle side is defined by $\left\{ {{\text{D}}_{{\text{n}}} \left( {\text{y}} \right),{\text{ y}},{\text{ f}}\left( {\text{y}} \right),{\text{ H}}} \right\};{\text{ASID}}_{{\text{i}}} = { }\left\{ {{\text{V}}_{{{\text{ID}}_{{\text{i}}} }} ,{\text{DSK}}_{{\text{i}}} } \right\}; D_{o} \left( {D_{{n_{i} }} \left( {m_{i} } \right)} \right)$ which can be computed as 704 bits; at the RSU side is defined by $\left\{ {{\text{RSU}}_{{{\text{ID}}_{{\text{i}}} }} ,{\text{S}}_{{{\text{RSU}}_{{\text{i}}} }} { }} \right\};$ $\left\{ {{\text{m}}_{{\text{i }}} ,{\text{ D}}_{{{\text{n}}_{{\text{i}}} }} \left( {{\text{m}}_{{\text{i}}} } \right)} \right\}$ as 256 bits and at the TTA/KGC/AS side is defined by $\left\{ {{\text{g}}_{{\text{i}}} ,{\text{kpvk}}_{{\text{i}}} ,{\text{DS}}_{{{\text{kpvk}}_{{\text{i}}} }} \left( {{\text{g}}_{{\text{i}}} ||\Delta {\text{T}}_{{\text{i}}} } \right)} \right\}$; $PSID_{{V_{i,n} }} = V_{{ID_{i} }} D_{{n \vartheta_{i,n} }} \left( y \right) mod \left( {f\left( y \right)} \right)$ is 384 bits. In a similar way, the storage cost is computed for the other existing schemes^23,25,27,29,30^. Table 11 provides the performance comparison in terms of Storage costs to the proposed Vs existing authentication schemes. Figure 8 provides the comparative analysis of storage costs of the proposed authentication scheme.

**Table 11:** Comparative Analysis on Storage Overhead.

Schemes	$V_{{ID_{i} }}$	RSU	TTA/KGC/AS	Total
^23^	868	868	2048	3784
^25^	512	–	768	1280
^27^	512	772	1280	2564
^29^	512q + 512	512q + 5122 + 256	256m + 256n + 256	1024q + 256m + 256n + 512r + 1024
^30^	668	1024	1536	2626
Proposed	704	256	384	1344

From the comparative analysis, it is evident that the proposed protocol incurs less computation overhead, communication and storage costs. In some cases due to higher number of cryptographic operations computation and communication costs is relatively high. This is mainly due to the increase in the number of bits for message transmission. A small compromise is needed in order to achieve more security requirements.

## Conclusion and future work

In this paper, we propose a conditional privacy preserving authentication and group key agreement authentication scheme for VANETs has been proposed. The proposed approach addressed the problem of computational burden in low-constrained resources of VANETs by using Dickson polynomial thereby optimizing the computing efficiency. By exploiting distributed approach, the scheme distributes the centralized role of TTA among KGC and AS thereby enhancing the robustness and countering the realistic demands of VANETs. Since the scheme employs distributed approach, the computational burden incurred by the TTA gets reduced thereby enabling group key agreement mutual authentication. The group key gets computed by the use of CRT which facilitates dynamic distribution and updation easily. Conditional privacy preservation can be achieved by the use of pseudonyms thereby assuring traceability and revocability. Since the group key is computed by means of distributed group key computation it provides enough security which is resilient towards TPD, ESL and Side-channel attacks. The scheme achieves traceability with the help of pseudonyms. The security strengths are proved using BAN, ROR model and Coq formal security verification tools and proofs are provided thereby demonstrating the resilience of the authentication scheme. Additionally a comparative analysis on security parameters, performance metrics has been analyzed with the existing schemes and is proven to be efficient than their counterparts. Finally the proposed protocol accomplishes the security and privacy properties with relatively low computation, communication and storage costs than the existing counterparts.

In the future work, distributed learning can be included where the scheme can be designed for data poisoning and free-rider attacks. Lattice and post quantum cryptographic approaches can be utilized to address the problems of security and privacy that benefits the low-resource constrained environment like VANETs.

## Data Availability

All data generated or analyzed during this study are included in this published article.
